# Microvesicles Derived from Adult Human Bone Marrow and Tissue Specific Mesenchymal Stem Cells Shuttle Selected Pattern of miRNAs

**DOI:** 10.1371/journal.pone.0011803

**Published:** 2010-07-27

**Authors:** Federica Collino, Maria Chiara Deregibus, Stefania Bruno, Luca Sterpone, Giulia Aghemo, Laura Viltono, Ciro Tetta, Giovanni Camussi

**Affiliations:** 1 Department of Internal Medicine and Center for Molecular Biotechnology, University of Torino, Torino, Italy; 2 Department of Automatic and Informatics, Politecnico, Torino, Italy; 3 SiS-Ter S.p.A., Palazzo Pignano, Crema, Italy; 4 Fresenius Medical Care, Bad Homburg, Germany; Harvard Medical School, United States of America

## Abstract

**Background:**

Cell-derived microvesicles (MVs) have been described as a new mechanism of cell-to-cell communication. MVs after internalization within target cells may deliver genetic information. Human bone marrow derived mesenchymal stem cells (MSCs) and liver resident stem cells (HLSCs) were shown to release MVs shuttling functional mRNAs. The aim of the present study was to evaluate whether MVs derived from MSCs and HLSCs contained selected micro-RNAs (miRNAs).

**Methodology/Principal Findings:**

MVs were isolated from MSCs and HLSCs. The presence in MVs of selected ribonucleoproteins involved in the traffic and stabilization of RNA was evaluated. We observed that MVs contained TIA, TIAR and HuR multifunctional proteins expressed in nuclei and stress granules, Stau1 and 2 implicated in the transport and stability of mRNA and Ago2 involved in miRNA transport and processing. RNA extracted from MVs and cells of origin was profiled for 365 known human mature miRNAs by real time PCR. Hierarchical clustering and similarity analysis of miRNAs showed 41 co-expressed miRNAs in MVs and cells. Some miRNAs were accumulated within MVs and absent in the cells after MV release; others were retained within the cells and not secreted in MVs. Gene ontology analysis of predicted and validated targets showed that the high expressed miRNAs in cells and MVs could be involved in multi-organ development, cell survival and differentiation. Few selected miRNAs shuttled by MVs were also associated with the immune system regulation. The highly expressed miRNAs in MVs were transferred to target cells after MV incorporation.

**Conclusions:**

This study demonstrated that MVs contained ribonucleoproteins involved in the intracellular traffic of RNA and selected pattern of miRNAs, suggesting a dynamic regulation of RNA compartmentalization in MVs. The observation that MV-highly expressed miRNAs were transferred to target cells, rises the possibility that the biological effect of stem cells may, at least in part, depend on MV-shuttled miRNAs. Data generated from this study, stimulate further functional investigations on the predicted target genes and pathways involved in the biological effect of human adult stem cells.

## Introduction

Cell-to-cell communication is a refined system to ensure proper coordination among different cell types in tissues. Besides soluble factors, cell-derived microvesicles (MVs) have been described as a new mechanism of communication. MVs are plasma membrane-derived vesicles/exosomes released in the micro-environment by various cell types [1], [2] including stem cells and progenitors [3], [4]. Ratajczak *et al* [3] first described that MVs derived from embryonic stem cells (ESC) may reprogram hematopoietic progenitors by a mRNA-dependent mechanism. Quesenberry and Aliotta have recently suggested [5] that a continuous genetic modulation of cells through transfer of MVs may be involved in the continuum change in bone marrow stem cell phenotype. MV-mediated transfer of genetic information from injured cells to bone marrow-derived stem cells may reprogram their phenotype to acquire features of the injured tissue [6]. Conversely, MVs derived from stem cells may induce de-differentiation of cells survived to injury with a cell cycle re-entry that may allow tissue regeneration [7]. We, recently, demonstrated that MVs derived from bone marrow mesenchymal stem cells (MSCs) contribute to the repair of acute kidney injury by mRNA transfer [8]. Moreover, MVs derived from human liver stem cells (HLSCs), a population of mesenchymal stem cells showing a partial hepatic commitment [9], accelerated the morphological and functional recovery of liver in a model of 70% hepatectomy in rats [10]. It has been suggested that MVs, after internalization within target cells through surface-expressed ligands, may transfer not only proteins, bioactive lipids and mRNAs but also microRNAs (miRNAs) [2], [11].

miRNAs are a group of small (21–24nt) noncoding RNAs that function as post-transcriptional regulators of gene expression by either triggering mRNA cleavage or repressing translation [12], [13], [14]. Valadi described that exosomes derived from mast cells may shuttle specific subsets of miRNAs [15]. miRNAs have been also found in peripheral blood MVs of healthy individuals [16] and of patients with ovarian tumors [17]. Moreover, Yuan et al. showed that MVs derived from ESC contained abundant miRNAs that can be transferred to mouse embryonic fibroblasts in vitro [11]. In addition, the conditioned medium of human ESC-derived MSCs contained microparticles enriched in pre-miRNAs [18]. The mechanism of mRNA and miRNA compartmentalization within MVs has not been clarified. However it is possible that ribonucleoproteins, that mediate the fate of RNAs within the cells, could be involved.

The aim of the present study was to investigate whether ribonucleoproteins which are known to be responsible for the intracellular traffic and compartmentalization of RNAs, are present in MVs released from human MSCs and HLSCs. Moreover, we comparatively investigated the miRNA content of MVs derived from MSCs and HLSCs and the ability of MVs to transfer miRNAs in target cells.

Our study demonstrates the presence within MVs released from MSCs and HLSCs of selected ribonucleoproteins that may account for mRNA and miRNA storage. In addition, we found that MVs derived from these cells contained miRNAs specific of the cell of origin and may serve as a signature for adult stem cells/MVs. Some miRNAs were found to be selectively accumulated inside the released MVs suggesting an organized package of miRNAs. Moreover, miRNAs from MVs were transferred and accumulated into recipient cells, where they may downregulate specific targets.

## Results

### MVs shuttle ribonucleoproteins involved in RNA traffic

**Figure 1:**
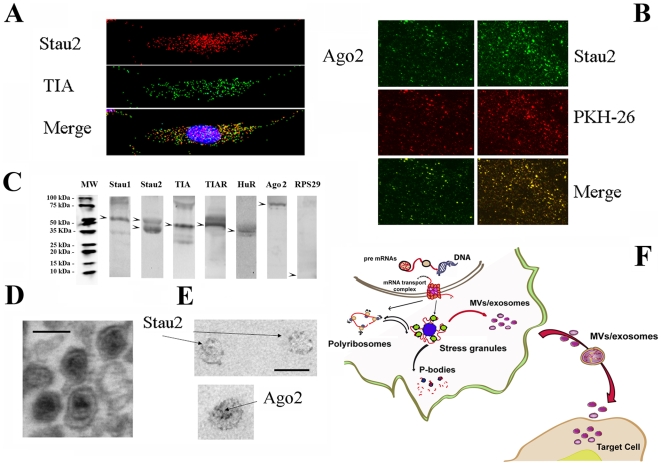
Expression of ribonucleoproteins within MVs. Panel A. Representative micrographs showing the presence of stress granule expressing Stau2 and TIA ribonucleoproteins as detected by confocal microscopy in MSCs cultured overnight in the absence of serum. Panel B: representative confocal micrographs showing the expression of Stau2 and Ago2 by MVs released from MSCs cultured overnight in the absence of serum. MVs were labeled with the red PKH26 dye. Merge shows the co-localization of Ago2 and Stau2 within MVs. MVs from MSCs expressed also Stau1, TIA, TIAR and HuR (not shown). (Panel A; original magnification X400. Panel B; original magnification X600). Panel C: representative western blot analysis showing the expression of Stau1, Stau2, TIA, TIAR, HuR, Ago2 and RPS29 by MVs derived from MSCs. Head arrows indicate the expected molecular weight. Panel D: Representative micrographs of transmission electron microscopy obtained on purified MVs. Ultrathin sections, stained with lead citrate. Panel E: Immunogold electron microscopy showing staining for Stau2 and Ago2 (see Methods). MVs were viewed by JEOL Jem 1010 electron microscope (black line = 100 nm). All experiments were performed three times with similar results. Panel F: schematic representation of ribonucleoprotein mediated RNA intracellular traffic, suggesting that MVs/exosomes may represent a site of RNA compartmentalization allowing the transfer of genetic material to target cells.

We previously demonstrated that MVs derived from MSCs and HLSCs contained selective patterns of mRNA (microarray data were deposited on GEO database as geo accession GSE 12243 and GSE15569 respectively). To investigate the mechanism of RNA transport by MVs, we evaluated the presence in MVs of selected ribonucleoproteins involved in the traffic of RNAs from nucleus to cytoplasm and in the RNA stabilization. As shown in figure 1, MVs derived from MSCs contained ribonucleoproteins as seen by Western blot, immunofluorescence and immunogold electron-microscopy. MVs contained T cell internal antigen-1 (TIA), TIA-1-related (TIAR) and AU-rich element binding protein (HuR), multifunctional proteins expressed in nuclei and stress granules [19], and proteins involved in the transport and stability of mRNA such as staufen1 (Stau1) and 2 (Stau2) [20]. Similar results were obtained with MVs derived from HLSCs (not shown). In addition, MVs derived from MSCs contained argonaute2 (Ago2), a protein of the argonaute family which is involved in miRNA transport and processing [21]. At variance with stress granules, MVs did not contain the human ribosomal protein S29 (RPS29) (Figure 1C). These results suggest that the RNA content of MVs released from MSCs and HLSCs was modulated by the same ribonucleoproteins involved in the intracellular trafficking of RNA among nucleus, polyribosomes, stress granules, P bodies and MVs/exosomes (Figure 1F).

### Cytoskeleton is involved in the release of MVs

**Figure 2:**
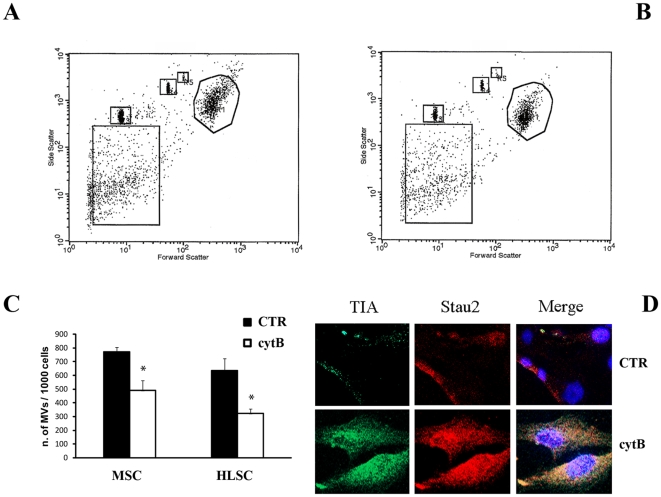
Effect of cytochalasin B on MV release and stress granule accumulation. Panel A and B: The release of MVs by MSCs (A) and HLSCs (B) was evaluated using the gating strategy described by Wysoczynsk [19]. Size beads were used to define the proper gate of MVs, as events under 1 µm. The stopping gate was set up on 1,000 events collected from the cells region (R1). Panel C: The number of MVs was evaluated by flow cytometry at 6 h for MSCs and 24 hours for HLSCs after 1 hour treatment with cytochalasin B (white bar, cyt B) or with vehicle alone (black bar, CTR). Data are expressed as mean ± 1SD of 5 experiments. Nonparametric Mann-Whitney t test  =  * p<0.05. Panel D: representative confocal micrographs of TIA and Stau2 expression by stress granules in MSCs pre-treated with cytochalasin B (cyt B) or vehicle (CTR) as described in Methods. Five experiments were performed with similar results.

To analyze the involvement of actin polymerization and cytoskeleton activation in MV release, we evaluated the number of MVs secreted by MSCs and HLSCs after cell pre-treatment with an inhibitor of actin polymerization, cytochalasin B [22]. Pre-treatment with 10 µg/ml of cytochalasin B, significantly reduced the MV release from MSCs and HLSCs (Figure 2A-C). Moreover, the inhibition of MV release by cytochalasin B was associated with an enhanced expression of stress granules within the cells, detected by the accumulation of TIA and Stau2 granules (Figure 2D).

### Profiling of miRNAs in MSCs and HLSCs

We performed comparative miRNA expression profiling analyses of 365 human mature miRNAs in two different lines of MSCs and HLSCs. We analyzed only the miRNAs similarly modulated in the two preparations of MSCs and HLSCs, respectively to focus on maintained miRNA patterns. Despite a qualitative difference, the number of miRNAs expressed by MSCs and HLSCs was similar, i.e. 145 and 139 mature miRNAs. The expression of the small nuclear RNAs (snoRNAs) used as internal controls was similar in MSCs (RNU6b: 32.5±1, RNU48: 23.9±0.2 and RNU44: 22.9±0.5, raw cycle threshold values, Ct) and HLSCs (RNU6b: 32.8±1.4, RNU48: 24.3±0.2 and RNU44: 22.6±0.2, raw Ct values), confirming the correct setting of the experiments. To reduce sample variations among different arrays, miRNAs were compared between MSC and HLSC samples, based on their cycle threshold (Ct) values normalized to the mean expression value, calculated on the overall miRNA expression in each array according to Mestdagh [23]. Normalized MSC and HLSC data were then subjected to hierarchical clustering analysis to compare their miRNA expression. In Table 1, miRNA expression was analyzed separately in MSCs and HLSCs to determine the most expressed miRNAs by both cell types. MSCs and HLSCs shared a common pattern of 9 miRNAs which were highly expressed in both cell types (Table 1). Moreover, MSCs expressed higher level of miR-21 than HLSCs whereas HLSCs expressed higher level of miR-19b than MSCs.

**Table 1:** Normalized expression analysis of miRNAs in MSCs and HLSCs.

MSCs		HLSCs	
miRNAs	Normalized Expression ±S.D.	miRNAs	Normalized Expression ±S.D.
**hsa-miR-125b**	157.2±44.7	**hsa-miR-24**	52.5±12.8
**hsa-miR-222**	120.1±10.7	**hsa-miR-222**	48.9±18.8
**hsa-miR-24**	70.9±25.4	**hsa-miR-99a**	43.3±7.5
**hsa-miR-99a**	67.9±5.4	**hsa-miR-125b**	37.8±0.2
**hsa-miR-100**	62.6±0.0	**hsa-miR-100**	37.2±0.3
**hsa-miR-594**	40.3±3.2	**hsa-miR-31**	30.9±12.3
**hsa-miR-31**	33.2±8.4	**hsa-miR-19b**	25.3±4.3
**hsa-miR-16**	29.4±3.1	**hsa-miR-16**	21.4±3.4
**hsa-miR-125a**	29.3±1.4	**hsa-miR-594**	18.8±4.2
**hsa-miR-21**	20.4±2.7	**hsa-miR-125a**	16.7±3.5

Expression of miRNAs in MSCs and HLSCs was analyzed separately (n = 4). miRNAs were clustered in each sample and compared intra-sample to detected conservative miRNAs inside the MSCs and HLSCs. Normalized miRNA expression was generated using the mean expression value normalization then the mean Ct was converted into expression (2^−ΔCt^) ± SD. The top ten highly expressed miRNAs in MSCs and HLSCs are reported.

**Figure 3:**
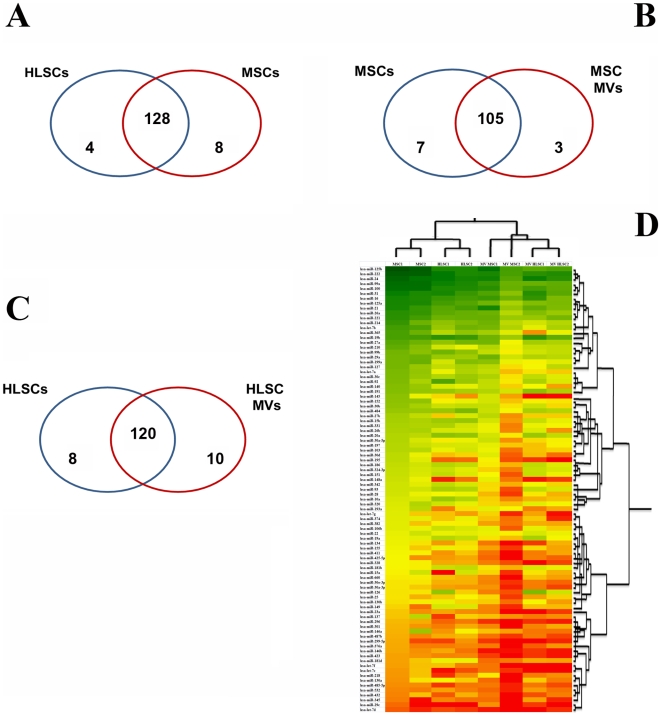
Venn diagram comparing miRNA expression in HLSCs, MSCs and their MVs and expression profile of common miRNAs. Panel A–C: the number of shared and specific miRNAs for MSCs and HLSCs (A), for MSCs and MSC MVs (B) and for HLSCs and HLSC MVs (C) are shown. Panel D: Heat map demonstrating the expression profile for MSCs, HLSCs and the corresponding MVs is generated for commonly expressed miRNAs.

To compare miRNA profile of MSCs and HLSCs, we performed clustering similarities between these two cell types, to detected groups of conservative or differentially modulated miRNAs. Normalized expression data were reanalyzed generating 3 different groups of miRNA expression for MSCs and HLSCs (see Methods). MSCs and HLSCs shared 128 miRNAs (Figure 3A), 98 of which clustered in the same expression group, suggesting a common origin of the two populations. Moreover, they showed 4 miRNAs selectively expressed by HLSCs (miR-7, -95, -204 and -650) and 8 by MSCs (miR-196b, -196a, -615, -501, -449, -17-3p, -497 and-486) (Table 2). Among miRNAs expressed by both cell types that clustered in same expression groups, we found that 32 miRNAs were more than 2-fold increased in MSCs compared to HLSCs and 3 miRNAs in HLSCs in respect to MSCs (Table S1), possibly reflecting the influence of the local niche in establishing specific miRNA patterns.

**Table 2:** miRNAs selectively expressed by MSCs and HLSCs.

miRNAs			
Expression in HLSCs	Fold change	Expression in MSCs	Fold change
**hsa-miR-650**	30.8±4.8	**hsa-miR-196b**	1324.6±416.2
**hsa-miR-95**	30.3±11.7	**hsa-miR-196a**	731.7±0.95
**hsa-miR-7**	11±7.75	**hsa-miR-615**	606.2±145.6
**hsa-miR-204**	8.7±5.5	**hsa-miR-501**	123.5±89.2
		**hsa-miR-449**	24.6±14.3
		**hsa-miR-17-3p**	18.1±12.1
		**hsa-miR-497**	8.5±0.6
		**hsa-miR-486**	6.5±0.7

Based on the analysis of clustering similarities on all cell samples, miRNAs present only in one of the two cell types, were defined as selectively expressed by MSCs or HLSCs. Based on this model miRNAs were classified to determine the different pattern of miRNAs detected in both cell types. Fold change was measured based on the normalized miRNA mean differences (2^−ΔCt^) ± SD, between cell samples.

### MVs from MSCs and HLSCs are enriched of mature miRNAs

**Figure 4:**
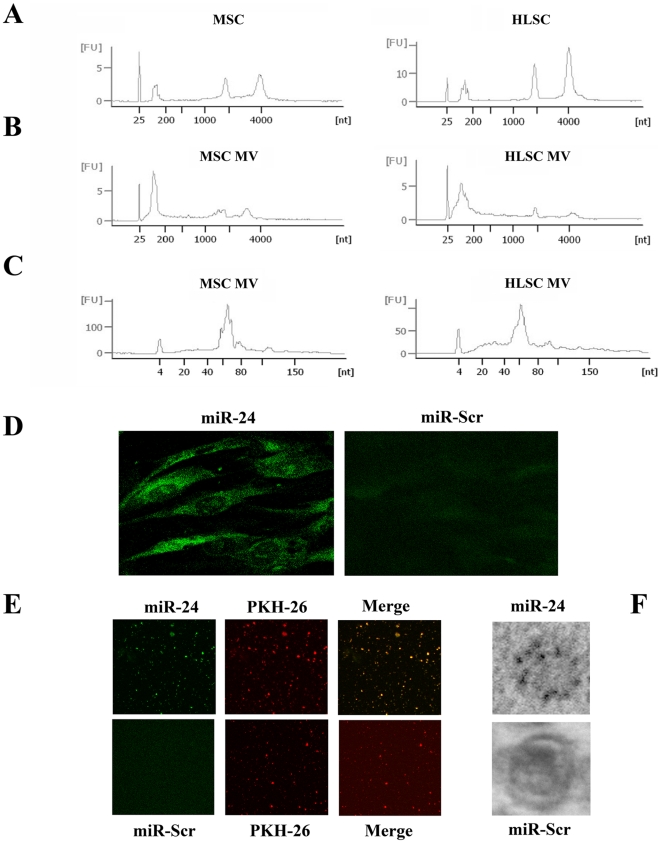
Bioanalyzer profile of RNA extracted from MSCs, HLSCs and the corresponding MVs and miRNA expression seen by in situ hybridization in cells and MVs. Panel A and B: Representative bioanalyzer profile of the RNAs contained in MSCs, HLSCs and MVs showing that, whereas the ribosomal subunit 28 and 18S were detectable in cells, they were absent or barely detectable in the corresponding MVs. MVs exhibited a relevant peak of small RNAs at variance with the cells. Panel C: Representative bioanalyzer profile of small RNAs performed on MVs derived from MSCs and HLSCs (MSC MVs and HLSC MVs) showing an enrichment of small RNAs (range: 30–50%) of the size of miRNAs in respect to the cells of origin (range: 4–8%; not shown). Three different samples tested in triplicate were analyzed for each type of cells and MVs with similar results. Panel D–F: Representative micrographs of in situ hybridization on MSCs (D) and MSC-derived MVs (E and F) using a probe for miR-24 or a scramble-miR probe (miR-Scr) as control. Confocal microscopy original magnification X400 (D) and X600 (E). In panel E, MVs were labeled with a red dye PKH26. Panel F: The in situ hybridization was revealed by immunogold transmission electron microscopy as described in Methods (original magnification X50,000). Three different experiments were performed with similar results.

We performed the same miRNA profiling of MVs purified from MSCs and HLSCs to test the hypothesis that MVs are enriched of mature miRNAs. A bioanalyzer profile on total RNA from MVs and their cells of origin revealed that MVs contained a broad range of RNA sizes, with a relevant peak characteristic of small RNA classes (Figure 4A and B). The enrichment in miRNAs was confirmed by the high percentage of the small RNAs detected in MVs that fell within the miRNA range of 10–40 nucleotides (48% in HLSC MVs and 32% in MSC MVs) (Figure 4C).

miRNA profile was conducted on these samples after Ct raw data normalization [23]. The content of control snoRNAs, RNU48 and RNU44, in MVs was significantly lower than that present within the cells of origin as previously shown for different mRNAs [8], [11]. For this reason, RNU48 and RNU44 were not used to normalize miRNA expression in MVs, instead a normalization analysis was performed using mean expression values derived from the overall miRNA expressed in each array [23]. Normalized MV and cell filtered data were then subjected to hierarchical clustering analysis to compare their miRNA expression generating clusters for each sample that were compared between all the cells and MVs or between cells and the corresponding MVs. We detected the presence of 113 mature miRNAs expressed by MSC MVs and 133 by HLSC MVs. Figure 3 underlines the similarities observed among MSCs, HLSCs and their MVs based on the relative miRNA expression. In particular, we found 91 miRNAs detected in both MVs and cells, showing the same pattern between MVs and their cell of origin but different level of miRNA expression. Among miRNAs expressed by both cells and MVs only 41 clustered in same expression groups (see Methods). Table 3 shows the miRNAs shared by MVs and their cells of origin that represented the most abundant miRNAs conserved in the cells and transported in MVs. The presence of miR-24 highly expressed by both cell types and MVs was confirmed by *in situ* hybridization that showed the accumulation of miR-24 inside MSCs as a punctuate pattern and in MSC MVs (Figure 4 D and E). Same results were obtained with HLSCs and HLSC MVs (not shown). Moreover, miR-24 accumulation on MVs was also detected by immunogold electron-microscopy as shown in figure 4F. We observed also that MVs expressed a group of miRNAs described as “selectively” expressed by MSCs [24], such as miR-103-1, -140, -143-5p and -340 (not shown).

**Table 3:** Expression analysis of miRNAs co-expressed by MVs and cells.

Normalized Expression ±S.D.				
miRNAs	MSCs	MSC MVs	HLSCs	HLSC MVs
**hsa-miR-125b**	296.2±82.55	66.1±57.6	51.3±0.25	20.4±3.15
**hsa-miR-222**	222.25±19.7	39.1±20.2	68.7±25.5	43.2±4.2
**hsa-miR-24**	135±46.85	40.75±29.6	72.3±17.4	54.4±18.5
**hsa-miR-99a**	125.7±9.9	23.7±14.1	59.1±10.2	15.0±1.8
**hsa-miR-100**	115.5±0.1	27.8±19	50.5±0.4	20.9±6.3
**hsa-miR-31**	62.2±15.5	14.6±9.4	43.55±16.7	30.55±17.6
**hsa-miR-16**	54.4±5.7	21.6±11.8	29.15±4.6	22.45±17.4
**hsa-miR-125a**	54.2±2.6	8.6±5.5	22.9±4.8	9.7±0.85
**hsa-miR-21**	43.7±15.6	31.9±35.7	22.2±4.7	12.5±3.8
**hsa-miR-221**	33.3±9.5	10.3±5.8	13.5±1.1	8.9±3.5
**hsa-miR-19b**	25.0±0.8	9.4±4.7	34.5±5.9	27.2±15.7

Expression of miRNAs in MSCs, HLSCs and their corresponding MVs was analyzed separately (n = 8), miRNAs were clustered based on their normalized mean expression value and compared inter-sample to detected conservative miRNAs inside the adult stem cells and their MVs. miRNAs classified in the first expression group are shown and expressed as (2^−ΔCt^) ± SD.

In addition, we were able to detect a group of miRNAs that resulted unique for MVs compared to the cells of origin whereas some miRNAs expressed by cells were absent in the corresponding MVs (Figure 3 B and C). In particular, 10 miRNAs were detected only in HLSC-derived MVs whereas 8 miRNAs were present in HLSCs and not in their MVs (Table 4). MSC-derived MVs expressed selectively only 3 miRNAs in respect to their cells of origin, instead 7 miRNAs were only detected in MSCs (Table 5).

**Table 4:** Selectively expressed miRNAs from HLSC MVs and their cells of origin.

miRNAs			
Expression in HLSCs	Fold change	Expression in HLSC MVs	Fold change
**hsa-miR-10b**	186.35±175.55	**hsa-miR-223**	1003.8±715.35
**hsa-miR-378**	96.1±76.8	**hsa-miR-142-3p**	437.1±43.95
**hsa-miR-95**	87.3±33.7	**hsa-miR-451**	403.7±164.8
**hsa-miR-432**	62.7±12.6	**hsa-miR-501**	324.4±179.2
**hsa-miR-642**	35.8±27.7	**hsa-miR-486**	61.7±8.4
**hsa-miR-143**	27.3±3.6	**hsa-miR-627**	59.6±42.9
**hsa-miR-199b**	10.1±5.4	**hsa-miR-142-5p**	59.1±8.4
**hsa-miR-502**	8.6±1.1	**hsa-miR-383**	16.05±8.4
		**hsa-miR-601**	7.2±2
		**hsa-miR-17-3p**	5.9±0.3

Expression of miRNAs in HLSCs and their corresponding MVs was analyzed separately (n = 4). Clustering similarities of MVs derived from HLSCs and their cells of origin detected miRNAs present only in HLSCs or their MVs. Data are expressed as fold change measured based on the normalized miRNA mean differences (2^−ΔCt^) ± SD, between HLSCs and their corresponding MVs.

**Table 5:** Selectively expressed miRNAs from MSC MVs and their cells of origin.

miRNAs			
Expression in MSC MVs	Fold change	Expression in MSCs	Fold change
**hsa-miR-223**	604±219.0	**hsa-miR-594**	714.4±56.3
**hsa-miR-451**	87.6±77.2	**hsa-miR-654**	322.25±227.8
**hsa-miR-564**	69.7±20.9	**hsa-miR-369-5p**	152.3±72.6
		**hsa-miR-502**	11.3±2.0
		**hsa-miR-376a**	11±3.2
		**hsa-miR-362**	6.3±0.7
		**hsa-miR-194**	2.6±0.6

Expression of miRNAs in MSCs and their corresponding MVs was analyzed separately (n = 4). Clustering similarities of MVs derived from MSCs and their cells of origin detected miRNAs present only in MSCs or their MVs. Data are expressed as fold change measured based on the normalized miRNA mean differences (2^−ΔCt^) ± SD, between MSCs and their corresponding MVs.

**Figure 5:**
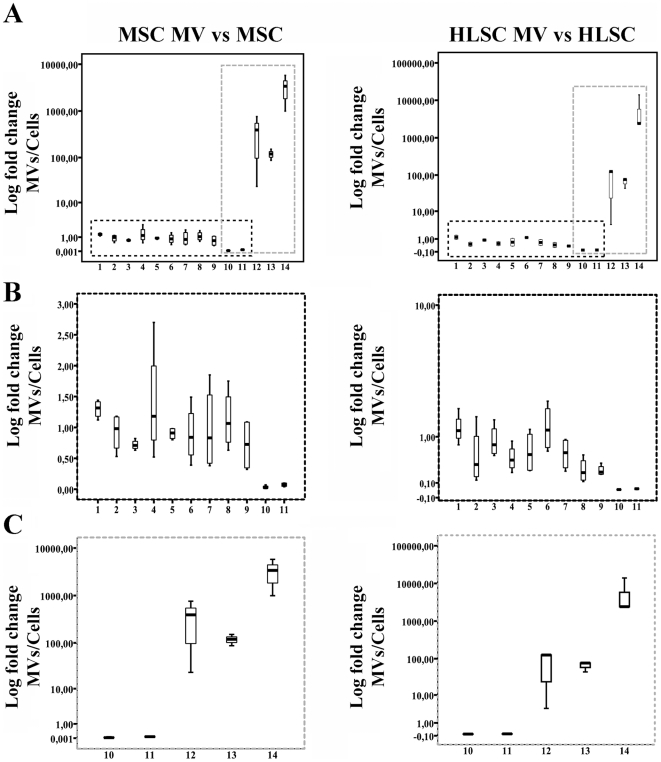
MVs containing miRNAs. Fold change analyses of selected miRNAs in MVs from MSCs and HLSCs in respect to their cell of origin was detected by qRT-PCR and expressed as Log of 2^−ΔCt^ (MVs versus cells). miRNA comparisons between cells and MVs were based on the relative expression data normalized using the geometric mean value of four of the most stable miRNAs identified in the profiling between cells and MVs (see Methods). The following miRNAs were tested: miR-221 (line1), miR-99a (line 2), miR-222 (line 3), miR-24 (line 4), miR-410 (line 5), miR-21 (line 6), miR-100 (line 7), miR-214 (line 8), miR-31 (line9), miR-223 (line 12), miR-122 (line 13) and miR-451 (line 14). As control snoRNAs, RNU 44 (line 10) and RNU 48 (line 11) were used. In panel A, the dark dotted box indicate the miRNAs co-expressed by MVs and the cells of origin and the grey dotted box indicate miRNAs enriched within MVs. Panel B and C are a magnification of the dark (B) and grey (C) boxes respectively. The boxed areas represent the mean ± quartile and the whiskers extend out to the minimum and maximum values. Anova with Newman-Keuls multicomparison test was performed and all the reported miRNAs exhibited a significance <0.001 in respect to the control snoRNAs.

As shown in figure 5, the relative abundance of several miRNAs in MVs derived from MSCs and HLSCs in respect to their cells of origin, was confirmed by quantitative real time-PCR (qRT-PCR) using the Syber green technique, supporting the data obtained by the MicroRNA Assay Panel. In particular, we observed that 9 miRNAs were co-expressed and 3 enriched in MVs from both MSCs and HLSCs (Figure 5). The finding that miRNAs at variance with mRNAs exhibited the same level or were even enriched within MVs suggests the compartmentalization of selected miRNAs in MVs before their release.

### Cell processes over-represented by target genes associated with the MV-miRNAs

**Figure 6:**
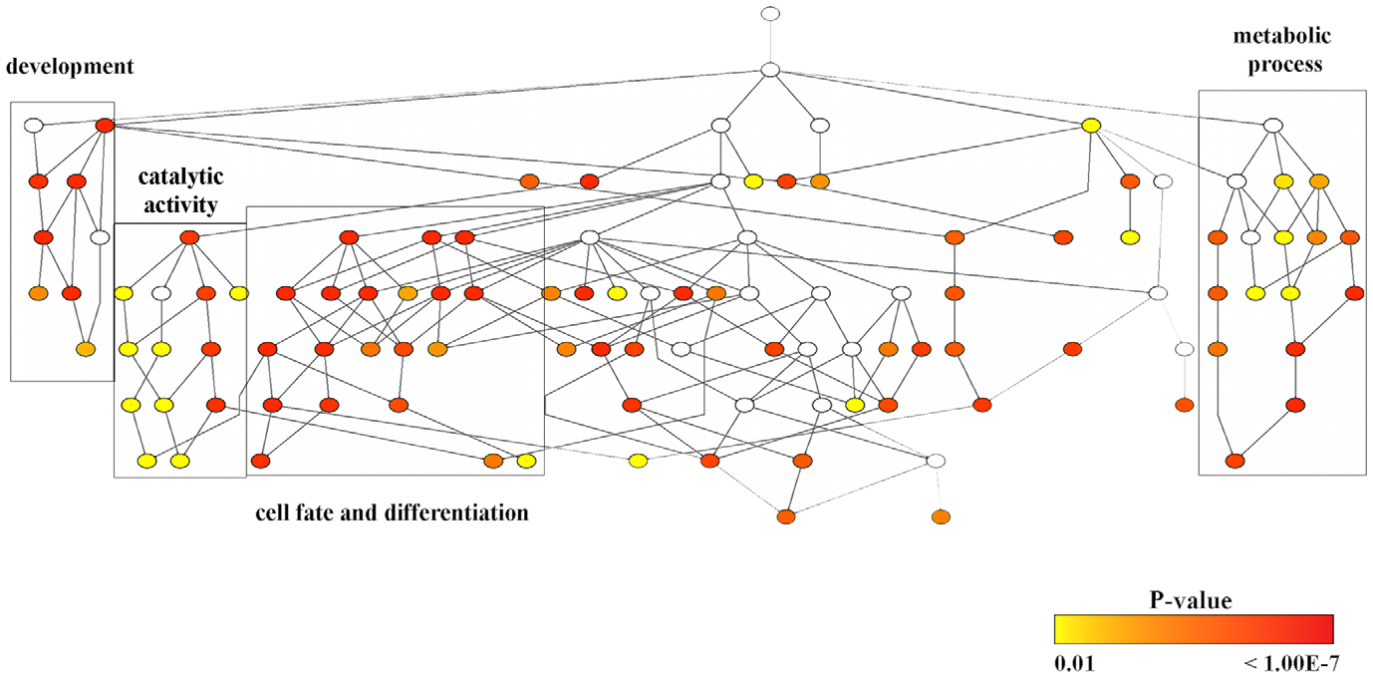
Cellular processes overrepresented by validated and predicted mRNA targets of miRNAs co-expressed by MVs and cells. A GO tree obtained from BiNGO, showing the hierarchy of gene ontology biological processes overrepresented by the validated and predicted targets of miRNAs co-expressed by MVs and cells. (p<0.01, see the color bar). Node colors represent the statistical significance. The white nodes (p>0.01) showed the relationships among their downstream nodes. The boxes indicated the overrepresented biological processes categorized into development, catalytic activity, cell fate differentiation and metabolic processes.

To characterize the cell processes modulated by highly expressed miRNAs, we analyzed the potential targets associated with the most abundant miRNAs conserved in the cells and transported by MVs (Table 3). Using the miRWalk database (see Methods), we detected 966 validated and 1,075 predicted genes associated with the highly co-expressed miRNAs in both cells and MVs. To characterize the biological processes overrepresented by these targets, we generated a network derived from the union of validated and predicted genes and we performed a gene ontology (GO) enrichment analysis using BiNGO 2.3 [25], a Cytoscape [26] plugin. Among the validated and predicted targets, 766 and 810 genes respectively, showed a GO annotation. In figure 6, it is shown a GO tree representing a hierarchical structure of the GO biological processes, in which the colored nodes indicate significantly overrepresented GO biological functions (*p*<0.01). We were able to identify in the overrepresented processes, four different groups: metabolic processes, developmental processes, catalytic activity and cell fate and differentiation. Among these groups, stem cell-related functions were detected, in particular development and differentiation-related functions, cell cycle-, proliferation- and cell death program-related processes were present (Table S2). Interestingly, these observations suggest that MV- and cell-enriched miRNAs can contribute to promoting cell proliferation/differentiation in target cells. Despite the similarities at the global level between the MV- and cell-derived miRNAs, we identified a group of miRNAs selectively expressed by MVs. The same GO analysis was conducted on MV-enriched miRNAs, to analyze the potential targets selectively regulated by the most abundant miRNAs transported in MVs but absent in cells after MV release. HLSC MV-enriched miRNAs (Table 4) resulted associated with 75 GO annotated genes (validated). Instead, MSC MV-enriched miRNAs (Table 5) were related to 42 validated target genes with a GO annotation. Interestingly, for both types of MV-enriched miRNAs, the GO biological processes that resulted highly represented were associated to metabolism, such as macromolecule metabolic processes and positive regulator of metabolic processes of RNA and DNA (*p* value: MSC MVs, 5.73E-12 and 4.61E-11; HLSC MVs, 5.89E-17 and 1.86E-16 respectively) (Table S3 and S4). Moreover, development related GO functions, including multicellular organism development, system and organ development and cell differentiation, assumed a relevant role in MV-enriched miRNAs GO classification in both MV types. Gene expression related processes such as transcription regulation and biosynthetic functions such as macromolecule biosynthesis (Table S3) were overrepresented in HLSC-derived MVs (19 and 20 out of 75 genes; *p* value: 9.06E-16 and 6.50 E-16 respectively). Surprisingly, MSC-derived MV selected miRNAs were associated to targets involved in the immune system processes, including leukocyte activation and differentiation and hemopoiesis regulation (Table S4).

### MSC-derived MVs are incorporated by murine tubular epithelial cells and transfer miRNAs

**Figure 7:**
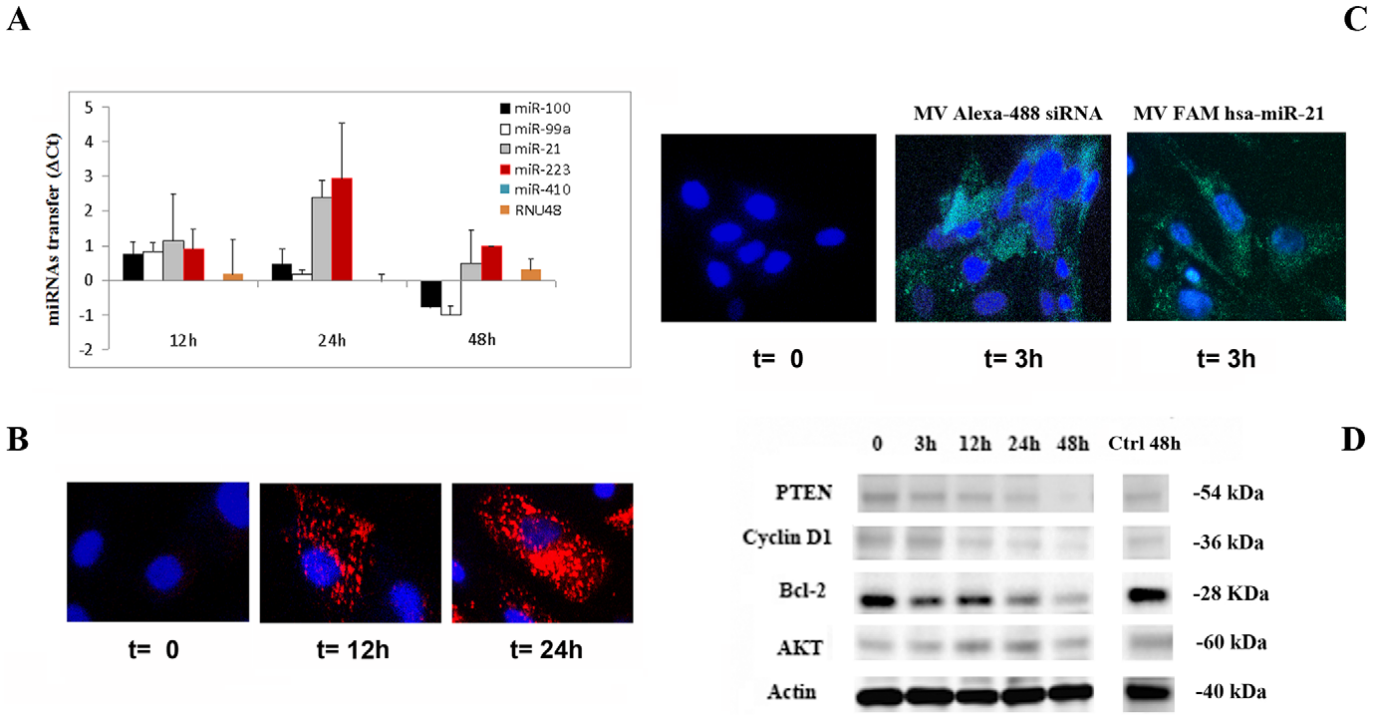
miRNA transfer and MV uptake by mTEC. Panel A: mTEC were incubated with MVs derived from MSCs for 12, 24 and 48 hours (h) at 37°C in the presence of 50 µg/ml α-amanitin to inhibit transcriptional activation in mTEC and the transfer of selected miRNAs was evaluated by qRT-PCR. The difference in Ct values between α-amanitin treated mTEC alone or stimulated with MVs is shown for each miRNA. The snoRNA, RNU48 and the miR-410, that was not present in MVs, were used as controls. Data are the mean ± SD of four experiments. Panel B: Micrograph representative of the incorporation of MVs labeled with PKH26 in mTEC evaluated by confocal microscopy after incubation for 12 and 24 hours at 37°C. Three experiments were performed with similar results (original magnification X400). Panel C: To evaluate the transfer of miRNAs within mTEC we used two reporter miRNAs, the Alexa-488 labeled siRNA or the FAM labeled microRNA Mimic (hsa-miR-21). mTEC were incubated for 3 hours at 37°C with MVs isolated from MSCs transfected with Alexa-488 labeled siRNA (MV Alexa-488 siRNA) or with FAM labeled microRNA Mimic (MV FAM hsa-miR-21). The uptake of MVs was evaluated by confocal microscopy. miRNAs incorporated within mTEC were detected as green fluorescence signal of Alexa-488 or FAM fluorophores (Original magnification X400). Panel D: Downregulation in mTEC of proteins targeted by some of the enriched miRNAs present in MVs, was evaluated by western blot analysis. mTEC were incubated at 37°C with 30 µg/ml of MVs derived from MSCs for 3, 12, 24 and 48 hours and the cell lysates were submitted to Western Blot as described in Methods for detection of PTEN, cyclin D1, Bcl-2, AKT and as control Actin. mTEC incubated for 48 hours in the absence of MVs were also used as control (Ctrl 48 h). Cell viability evaluated as trypan blue exclusion was 98±0.7%. Three experiments were conducted with similar results.

The presence of enriched miRNAs in adult stem cell MVs prompted us to investigate whether MVs may transfer miRNAs to target cells. To test this hypothesis murine tubular epithelial cells (mTEC) were incubated with MVs derived from MSCs in the presence of α-amanitin to inhibit transcriptional activation [27] and the transfer of selected miRNAs was evaluated by qRT-PCR. The variation in Ct values in mTEC stimulated with MVs was evaluated in respect to mTEC treated with α-amanitin in the absence of MVs. The abundance of some miRNAs (miR-21, miR-100, miR-99a and miR-223) increased progressively in mTEC concomitantly with the internalization of PKH26- labeled MVs (Figure 7A and B). MV internalization and accumulation of miRNAs peaked between 12 and 24 hours to decrease at 48 hours. However, this was not observed with all miRNAs tested. The miRNAs transferred most efficiently were those found in abundance in MVs. The RNU48, low expressed in MV, was less accumulated than the most abundant miRNAs. miR-410, not present in MVs, was not transferred to mTEC (Figure 7A). This suggested that the increase in specific miRNA content was due to their transport from MVs to the target cells. Moreover, the transfer of miRNAs within mTEC was confirmed using two fluorescent labeled reporter miRNAs carried by MVs derived from MSCs transfected with Alexa-488 labeled siRNA or with FAM labeled miRNA Mimics (hsa-miR-21 and hsa-miR-100). As seen by confocal microscopy, mTEC showed after 3 hour incubation with MVs a fine granular fluorescent pattern within their cytoplasm indicating the incorporation of the reporter Alexa-488 siRNA or FAM hsa-miR-21 (Figure 7C). Similar results were obtained with FAM hsa-miR-100 (not shown). Moreover, the incubation of mTEC with MVs derived from MSCs resulted in the reduction of proteins known to be targeted by some of the enriched miRNAs found in MVs (Figure 7D). In particular, the following target proteins were downregulated: PTEN which is known to be targeted by miR-21 [28], [29], cyclin D1 which is known to be targeted by miR-100, miR-99a and miR-223 [30] and Bcl-2 which is known to be targeted directly by miR-34, miR-181b and miR-16 [31], [32], [33] or indirectly modulated by miR-21 [34]. No downregulation was observed for AKT and for actin, used as control.

## Discussion

The results of the present study demonstrated that MVs contained ribonucleoproteins involved in the intracellular traffic of RNAs suggesting a dynamic regulation of RNA compartmentalization in the MVs released from human adult stem cells of mesenchymal origin. Moreover, MVs derived from MSCs and HLSCs, contained selective patterns of miRNAs which may be transferred to target cells.

Cell communication represents a dynamic mechanism regulated by the release of factors able to influence cell fate, function and plasticity. Recently, MVs/exosomes have been described as a potent paracrine mechanism that may re-direct cell fate through the active transfer of proteins, functional mRNAs and miRNAs [7], [35].

MVs may be released from the endosomal compartment after fusion with the plasma membrane [36], [37] or as shedding vesicles after budding and detachment of small cytoplasmic protrusions [38]. MVs present in the circulation, are a heterogeneous population that differs in size, antigenic composition and in cellular origin [39]. In physiological conditions, MVs in blood are derived mainly from platelets [40], and in lesser extent from other blood cells and endothelial cells [41]. It is now recognized that MVs from different cells may be internalized in target cells through specific receptor-ligand interactions [42] and transfer signals that may functionally affect them. It has been recently shown that stem/progenitor cells are a potential source of MVs suggesting that they may play a role in stem cell biology [5]. The recent finding that MVs may carry selected patterns of mRNAs and miRNAs suggests that MVs may represent a new mechanism of genetic exchange between cells [3], [4], [11], [43]. Ratajczak et al. [3] demonstrated that MVs released from ESC may reprogram hematopoietic progenitors by delivering mRNAs and proteins. We demonstrated that MVs from endothelial progenitor cells may activate in normal endothelial cells an angiogenic program by transfer of functional mRNAs [4]. Recently, it has been shown that MVs derived from ESC may transfer in target cells not only mRNAs but also mature miRNAs [11]. The mechanism of mRNA and miRNA compartmentalization and in particular whether RNAs are accumulated into MVs in a random or organized manner remain to be defined. However, recent studies [18], [44] as well as the present results suggest a selective mechanism of RNA packaging into MVs. Monitoring the ribonucleoproteins involved in the intracellular traffic of RNAs and comparing the species of miRNAs contained in MVs and cells of origin may provide information on the mechanism of RNA accumulation within MVs. We found that MVs released from MSCs and HLSCs contained stress granule specific proteins. In particular, MVs contained ribonucleoproteins involved in the RNA storage. TIA, TIAR and HuR have been detected inside MVs from both MSCs and HLSCs, together with Stau 1 and 2, proteins expressed in nuclei and stress granules but not in P-bodies [45]. The absence of P-body specific proteins was also observed in exosomes released from cultured monocytes which however contained proteins involved in miRNA organization such as Ago2 and GW182 [44]. We also observed the presence of Ago2 in MVs derived from MSCs. These data suggest a role for ribonucleoproteins in the RNA transport, storage and stability within MVs. At variance of stress granules [45], MVs did not contain the ribosomal protein, RPS29. The secretion of MVs was shown to be dependent on cytoskeleton activation as cytochalasin B inhibited their release and favored the accumulation of stress granules within the cells as detected by the increase of stress granule specific proteins, TIA and Stau2, inside MSCs treated with cytochalasin B. This observation is consistent with the previously reported enhancement of stress granules in cells treated with cytochalasin B [46]. Taken together these results suggest that ribonucleoproteins may shuttle RNAs from different cytoplasmic compartments including MVs/exosomes [47] possibly accounting for the specific accumulation of mRNAs and miRNAs in these structures.

miRNAs have been described as potential regulators of self renewal [48], plasticity [49] and differentiation potential of many stem cell types including MSCs [50], [51], [52]. This supports the theory that miRNA patterns may be used as signatures to define and track different cell populations [53], [54]. In the present study we adopted a two-phases approach consisting in a hierarchical clustering followed by a similarity analysis measuring the equalities or differences between miRNAs from MSCs, HLSCs and their MVs. With this approach, we defined a pattern of relevant miRNAs showing the same behavior among cells and MVs. The examination of predictive targets modulated by the most highly expressed miRNAs in MSCs and HLSCs and their MVs demonstrated that many of these miRNAs could be involved in multi-organ development, cell survival and differentiation. In fact, as reported herein, miRNA patterns may be used as signatures to define the cellular origin of MVs. Interestingly, 5 miRNAs selectively present in MSCs (see Table 2) were known to be expressed by bone marrow (mirWalk database), indicating a tissue specificity. Moreover, we confirmed that MVs derived from MSCs expressed a group of miRNAs specific for MSCs [24]. MSCs and their MVs co-expressed also miR-335, poorly detected in HLSCs and HLSC MVs, described as a specific bone marrow-derived MSC miRNA [24].

The comparative screening of miRNA content of MVs and adult stem cells may provide information on the mechanism of RNA accumulation within MVs. In particular, miRNA profile on MVs from MSCs and HLSCs showed that MVs contained a pattern of miRNAs shared with their cells of origin. However, selected miRNAs were detectable only in the released MVs but absent in the MSCs and HLSCs from which they derived supporting the theory of a specific and organized package of miRNAs in MVs before their secretion. When the miRNA content of HLSC MVs was compared with that of the cells of origin, miR-223, miR-142-3p and miR-451 represented the most highly differentially expressed miRNAs. When the miRNA content of MSC-derived MVs was compared with that of the cells of origin, miR-223, miR-564 and miR-451 represented the three selectively expressed miRNAs in MVs. Instead, miR-378, miR-10b and miR-95 were expressed mainly in HLSCs and miR-369-5p, miR-594 and miR-654, were expressed mainly in MSCs rather than in their MVs, suggesting that these miRNAs were not compartmentalized within MVs and therefore not secreted. Interestingly, both MVs from MSCs and HLSCs showed the selective expression of miR-451 and miR-223, not detected in their cells of origin after the MV release. Previous research indicated that miR-451 is involved in the specific differentiation of erythrocytes [55], [56]. However, high expression of miR-451 has been detected in other tissues, suggesting that numerous cell types may require miR-451 for their maintenance and/or differentiation [57]. Moreover, miR-451 deficiency has been associated to a poor prognosis in gastric cancers [58]. Interestingly, also miR-223 was predicted to influence cell differentiation and development [59], in particular miR-223 appears to have a role in hematopoietic stem cell proliferation [60]. A recent report showed that miR-223 was the most highly expressed miRNA in peripheral blood mononuclear cells, platelets and their plasma microvesicles [16]. Moreover, miR-223 has been also involved as regulator of cell cycle in cancer with an inverse correlation with cancer progression [61].

The presence of enriched miRNAs in adult stem cell MVs allowed us to demonstrate that MVs may transfer miRNAs to target cells. Indeed, we found that miRNAs highly expressed within MVs were transferred in mTEC after internalization of MVs. This observation is consistent with that of Yuan et al. [11] who demonstrated that miRNAs derived from ESC may be transferred to fibroblasts. Moreover, it has been described that specific miRNAs carried by apoptotic bodies are delivered to target cells and directly or indirectly regulate specific genes [62], [63]. Interestingly, we found that MVs may deliver to target cells not only endogenous miRNAs but also traceable synthetic miRNAs. Moreover, we found that miRNAs shuttled by MVs are functional because able to downregulate proteins such as Bcl-2, cyclin D1 and PTEN, known to be targeted by selected miRNAs. These data suggest that MVs may act as mediators of cell to cell communication also through miRNA delivery.

In conclusion, the results of the present study suggest the presence of a regulated mechanism of miRNA accumulation within MVs and their transfer to neighbor cells. The prediction of defined functions of miRNAs shuttled by MVs derived from adult stem cells suggests that they may be important factors in autocrine and paracrine regulation of development, differentiation and cell survival.

## Methods

### Isolation and Characterization of Human MSCs and HLSCs

Approval of the study was obtained from the Molecular Biotechnology Center (University of Torino) Institutional Review Board. MSCs were obtained from Lonza (Basel, Switzerland), cultured and characterized as previously described by Bruno [8].

HLSCs were isolated from human cryopreserved normal adult hepatocytes (Lonza). HLSC isolation, culture and characterization were performed as previously described [9]. MSCs and HLSCs were used within the sixth passage of culture. At each passage, cells were counted and analyzed by cytofluorimetric analysis and immunofluorescence to confirm their phenotype. Cells were characterized by FACS analysis for the expression of mesenchymal stem cell markers, and in the case of HLSCs also for the expression of tissue specific markers, as previously described [9]. Both cells were able to undergo osteogenic, adipogenic and chondrogenic differentiation when cultured in the appropriate differentiative media [8], [9]. HLSCs were also able to differentiate in mature hepatocytes [9].

### Isolation of MVs

MVs were obtained from supernatants of MSCs and of HLSCs as previously described [8], [10]. Briefly, MSCs were cultured in RPMI deprived of fetal calf serum (FCS) and supplemented with 0.5% of bovine serum albumin (BSA) (Sigma-Aldrich, St. Louis, MO). HLSCs were cultured overnight in α-MEM deprived of FCS. The viability of both cell types at the time of MV collection was >99% as detected by trypan blue exclusion. After centrifugation at 2,000 g for 20 minutes to remove debris, cell-free supernatants were ultracentrifuged at 100,000 g (Beckman Coulter Optima L-90K) for 1 hour at 4°C, washed in serum-free medium 199 containing Hepes 25 mM (Sigma) and submitted to a second ultracentrifugation in the same conditions [9]. The protein content of MV preparations was quantified by Bradford method (Bio-Rad, Hercules, CA, USA). In selected experiments, MVs were labeled with the red fluorescence aliphatic chromophore intercalating into lipid bilayers, PKH26 dye (Sigma). After labeling, MVs were washed and ultracentrifuged at 100,000 g for 1 h at 4°C. Endotoxin contamination of MVs was excluded by Limulus test (Charles River Laboratories, Inc., Wilmington, MA, USA) according to the manufacturer's instruction, and MVs were stored at –80°C. The analyses on MV suspension after staining with propidium iodide did not show the presence of apoptotic bodies.

### Cytofluorimetric quantification of MVs released from MSCs and HLSCs

MSCs and HLSCs were seeded in a 12-well tissue culture plate and cultured in their complete media until 80% of confluence. Cells were then incubated for 1 hour with vehicle (complete medium) or with 10 µg/ml of cytochalasin B (Sigma). After washing, culture media were replaced with serum-free media (0.5% of BSA) to induce MV release. Subsequently, 6 and 24 hours later, the number of secreted MVs in respect to the number of cells, was evaluated using flow cytometry by gating events smaller than 1 µm. Size beads (1–4–10 µm, Molecular Probes, Invitrogen, Carlsbad, CA) were used to establish the proper gate for events smaller than 1 µm, which include MVs. The stopping gate was set up on 1,000 events collected from the cell region [64]. Cell viability at the time of MV collection was >99% as detected by trypan blue exclusion.

### RNA extraction

Total RNA was isolated from different MV and cell preparations using the mirVana RNA isolation kit (Ambion) according to the manufacturer's protocol. RNA was then quantified spectrophotometrically (Nanodrop ND-1000, Wilmington DE) and the RNA quality was assessed by capillary electrophoresis on an Agilent 2100 Bioanalyzer (Agilent Technologies, Inc, Santa Clara, CA) where the presence of small RNAs was verified in both MV and cell samples. For RNA isolated from MSCs and HLSCs, only a RNA integrity number (RIN) ≥9 was used. Since the intact 18S and 28S rRNAs were scarcely detectable in the MVs, the RIN was not a constraint for these samples.

### miRNA profiling by quantitative real-time PCR

miRNA expression levels were analyzed using the Applied Biosystems TaqMan® MicroRNA Assay Human Panel Early Access kit (Applied Biosystems, Foster City, CA) to profile 365 mature miRNAs by qRT-PCR. The kit used gene-specific stem-loop reverse transcription primers and TaqMan probes to detect mature miRNA transcripts in a 2-step real-time reverse-transcription PCR assay. Briefly, single stranded cDNA was generated from total RNA sample (80 ng) by reverse transcription using a mixture of looped primers (Multiplex RT kit, Applied Biosystems) following manufacturer's protocol. The RT reactions were then diluted and mixed with a Taqman universal master Mix (Applied) in a ratio 1∶1, loaded in the TaqMan micro-fluid card and qRT-PCR experiments were performed. All reactions were performed using an Applied Biosystems 7900HT real-time PCR instrument equipped with a 384 well reaction plate. Raw Ct values were calculated using the SDS software version 2.3. using automatic baseline and threshold.

Another approach of qRT-PCR was used to confirm some miRNAs screened by microarray analysis. Briefly, 200 ng of input RNA from all samples was reverse transcribed with the miScript Reverse Transcription Kit and the cDNA was then used to detect and quantify miRNAs of interest by qRT-PCR using the miScript SYBR Green PCR Kit (all from Qiagen, Valencia, CA, USA). All samples were run in triplicate using 3 ng of cDNA for each reaction as described by the manufacturer's protocol (Qiagen). miRNAs specific primers to hsa-miR-221, -99a, -222, -24, -21, -100, -214, -31, -410, -16-1, -181b, -223, -122 and -451 were used in separate reactions. The snoRNAs, RNU48 and RNU44 were used as positive controls. Negative controls using 10 µl of water in place of the RNA were performed alongside each reaction. qRT-PCR was performed using a 48- well StepOne™ Real Time System (Applied Biosystems). miRNA comparisons between cells and MVs were performed on the relative expression data normalized using the geometric mean value of four of the most stable miRNAs identified in the profiling between cells and MV (miR-21, -221, -16-1, and 181b for HLSCs and HLSC MVs; miR-21, -221, -16-1, and 24 for MSCs and MSC MVs). Fold change in miRNA expression was calculated based on the normalized mean differences between cells and the corresponding MVs (2^−ΔCt^).

### Detection of RNA-binding proteins in cells and MVs

Indirect immunofluorescence was performed on cells cultured on chamber slides (Nalgen Nunc International, Rochester, NY) or PKH26-labeled MVs spotted on glass slides, fixed in 4% paraformaldheyde in PBS containing 2% sucrose for 15 min and permeabilized with cold methanol (−20°C) as described by Kedersha and Anderson [65]. After blocking with 1% BSA in PBS, samples were incubated with the following primary antibodies (1∶100 dilution) mouse anti-human Ago2 (AbCam, Cambridge Science Park, UK), goat anti-human TIA1, goat anti-human TIAR, mouse anti-human HuR (Santa Cruz Biotechnology, Santa Cruz CA), rabbit anti-human Stau1, mouse anti-human Stau2 (AbCam) and rabbit anti-human RPS29 (AbCam). After washings cells were incubated with the appropriate secondary antibodies at 1∶1,000 dilution (goat anti-mouse or donkey anti-goat labeled with Texas red or Alexa Fluor 488; Molecular Probe, Invitrogen). Cell nuclei were stained with DAPI (Sigma). Confocal microscopy analysis was performed using a Zeiss confocal microscope, model LSM 5 PASCAL (Carl Zeiss International, Germany).

### Electron microscopy of MVs

MVs from MSCs or HLSCs were suspended in PBS and loaded onto 200 mesh nickel formvar carbon coated grids (Electron Microscopy Science, Hatfield, PA). MVs were then fixed in 3% paraformaldheyde (PAF) in PBS containing 2% sucrose for 15 min and permeabilized with cold methanol (−20°C) for 10 min. After extensive washing and blocking with 1% BSA in PBS pH 7.4, MVs were immunolabeled with primary antibodies for 1 hour at room temperature and after washings with PBS/BSA (pH 8.2), MVs were incubated with 10 or 5 nm gold labeled goat anti-rabbit of anti-mouse antibodies (BBInternational, Cardiff, UK) in PBS/BSA pH 8.2 for 1 hour at room temperature. MVs were postfixed in 2.5% gluteraldehyde, washed and after silver enhancement (BBInternational), embedded in a mixture of uranyl acetate (0.8%) and methyl cellulose (0.13%), and examined in a Jeol JEM 1010 electron microscope.

### Western Blot Analysis

MVs and cells were lysed at 4°C for 1 hour in a lysis buffer (50 mM Tris-HCl, pH 8.3, containing 1% Triton X-100, 1 mM PMSF, 10 µg/ml leupeptin, and 100 units/ml aprotinin). Aliquots of the MV and cell lysates containing 30 µg of proteins, as determined by Bradford method, were subjected to 4–15% gradient SDS-PAGE under reducing conditions and electroblotted onto nitrocellulose membrane filters as previously described [4]. The blots were blocked with 5% non fat milk in 20 mM Tris-HCl pH 7.5, 500 mM NaCl plus 0.1% Tween (TBS-T). The MV membranes were subsequently immunoblotted at 4°C with relevant primary antibodies (anti-human TIA1, TIAR and HuR from Santa Cruz Biotechnology; anti-human Ago2, Stau1, Stau2 and RPS29 from AbCam) or irrelevant isotypic controls at appropriate concentration. The cell lysate membranes were subsequently immunoblotted at 4°C with relevant primary antibodies against cyclin D1 (AbCam), PTEN (Cell Signaling Technology, Danvers, MA, USA), Bcl-2 (Santa Cruz Biotechnology), AKT (Cell Signaling Technology) and β-actin (Santa Cruz Biotechnology). After extensive washings with TBS-T, the blots were incubated for one hour at room temperature with peroxydase conjugated secondary antibodies (Santa Cruz Biotechnology), washed with TBS-T, developed with ECL detection reagents (Amersham) and detected by Chemidoc (Biorad).

### In situ hybridization

*In situ* hybridization was conducted on HLSCs, MSCs and their MVs and performed using a miRCURY LNA detection probe against hsa-miR-24 (Exiqon, Vedbaek, Denmark), showing to be highly expressed by cells and MVs. The probe used was biotinylated at 5′ of its sequence and a scramble-miR probe (5′-biotin) was used as control. Protocol was conducted according to the manufacture instruction. Briefly, MVs were isolated and spotted on formvar-carbon coated grid for electron microscopy analysis. Cells were seeded into chamber slides (Nunc, Rochester, NY, United States) and incubated under normal growth conditions overnight, reaching 80% of confluence. Cells and MVs were then fixed with 4% PAF for 30 min at room temperature, washed three times with 1X PBS. For the in situ hybridization on cells, HLSCs and MSCs were permeabilized at 4°C in 70% ethanol overnight. Hybridization with the LNA probe (10 nM) was carried out at 20–22°C below the melting temperature of the probe overnight after incubation in a pre-hybridization buffer (50% Formamide, 5xSSC, 0.1%Tween, 9.2 mM citric acid for adjustment to pH 6, 50 µg/mL heparin, 500 µg/mL tRNAs) for 2 h at the temperature of annealing of the probe (59°C) in a water bath. Prior to incubate the samples, the LNA detection probe was warmed in the hybridization buffer at 80°C to linearize the probe. The day after the samples were rinsed in stringent conditions (50% formamide, 2× SSC at hybridization temperature), washed for 1 h with a blocking solution (1% BSA in PBS-T) at room temperature followed by an incubation with 10 nm gold-conjugated avidin (BBInternational) and a silver enhancement (Silver enhancing kit, BBInternational). Grids were then counterstained and examined with a Jeol JEM 1010 electron microscope. For the immunofluorescence detection, cells and MVs were incubated with a Atto-488 conjugated streptavidin (Sigma) and analyzed by confocal microscopy.

### Transient transfection assay

Transfections of siRNA and miRNAs to MSCs were performed using the HiPerFect Transfection Reagent (Qiagen) according to the manufacturer's protocol. AllStars Negative Control siRNA Alexa 488 (Qiagen, 100 nM) and FAM conjugated MISSION® microRNA Mimics (Sigma, hsa-miR-100 and hsa-miR-21, 100 nM) were used in each experiment.

### Transfer of miRNAs to murine tubular epithelial cells

To analyze miRNAs transfer from MVs, mTEC were used. mTEC were isolated, cultured and characterized as previously described [8]. miRNA transfer experiments were conducted as previously by Yuan [11]. Approximately 5×10^5^ cells/well of mTECs were pre-plated in a 65 cm^2^ Petri dish, 1 day before the stimulation. MSC MVs isolated from 8 T175 flasks were used for each experiment and equally divided between all mTEC samples. mTEC were co-incubated for different times with MSC MVs and a transcription inhibitor, α-amanitin (Sigma, 50 µg/ml) or with α-amanitin alone [27] to inhibit transcriptional activation induced by MVs. Cells were washed with PBS twice, to eliminate any residual of MSC MVs, then enzymatically dissociated from the plates and washed with PBS. Cells were collected at time 0 and after 12, 24 and 48 hours of stimulation with MVs plus α-amanitin or with α-amanitin alone as control. Total RNA from mTEC was isolated and qRT-PCR was performed for a subset of miRNAs using the protocol described above. As an indirect measure of miRNA transfer, we determined the difference in Ct values between α-amanitin treated cells in the absence or in the presence of MSC MVs at each experimental time point; a positive value indicated transfer. If no signal was detected, a Ct value of 40 was assigned to the sample. Moreover, to study the kinetic of MV incorporation into mTEC, we incubated 10 µg of MVs, labeled with PKH26 dye for 12 and 24 hours at 37°C. For miRNA transfer studies, mTEC were also incubated for 3 hours at 37°C with MVs isolated from siRNA-Alexa 488 or FAM-microRNA Mimic transfected MSCs. MV uptake was evaluated for both the experiments by confocal microscopy.

### Clustering of miRNAs and similarity analyses

We adopted a two-phases approach consisting in a hierarchical clustering followed by a similarity analysis. Raw data were normalized considering the average Ct computed for each clustering phase, and then subjected to hierarchical clustering using a mean-centering data grouping miRNAs with a Ct value mean difference lower than 1.2. Six miRNA groups were identified between the miRNA expression patterns of each assay. Groups generated from hierarchical clustering were then compared between different sample through the similarity analyses based on three miRNAs expression conditions, highly expressed (normalized expression value lower than 0), expressed (normalized expression value greater than 0) or not expressed. The process identified several contingency tables where each miRNA was classified in several categories that allowed identifying its behavior [66]. Two similarity categories were defined: equally expressed, where the included miRNAs were highly expressed, expressed or not expressed; or differentially expressed, where the included miRNAs had a complementary behavior between the considered assays (less expressed to more expressed or vice versa). For both the categories, we compared miRNA behavior between MSCs and HLSCs, between all the cells and MVs or between cells and the corresponding MVs, generating also sub-categories including only MSCs, HLSCs or their corresponding MVs, to identify biological relevant miRNAs.

### Statistical analysis

Since in qRT-PCR, Ct scores greater than 35 are considered non-specific [67], miRNAs that had a raw Ct value greater than 35, conserved in both preparations of MSCs, HLSCs or their corresponding MVs, were not included in the final data analysis. Using these filtering criteria, we removed from analysis, 187 miRNAs for MSCs, 195 for HLSCs, 199 for MSC MVs and 208 for HLSC MVs, respectively. The internal controls (snoRNAs) in MVs were significantly different from that of their cells of origin. Relative expression data were then normalized using the mean expression value, calculated on the overall miRNA expression in each array, according to a Ct detection cut-off of 35 PCR cycles as described by Mestdagh et al [23]. The mean expression value stability as normalizer was confirmed by geNorm analysis [23]. Fold change in miRNA expression was calculated based on the normalized mean differences between cells, cells and MVs or between cells and the corresponding MVs (2^−ΔCt^).

Statistical analysis was performed by using the t test or ANOVA with Newman-Keuls multicomparison test, where appropriate. A p value of <0.05 was considered significant.

### Pathway analysis and prediction

Validated and predicted target genes for MV-miRNAs were obtained from the online database, miRWalk (http://www.ma.uni-heidelberg.de/apps/zmf/mirwalk). mirWalk miRNA target prediction was provided by the match among four different miRNA prediction programs (miRanda, miRDB, miRWalk and TargetScan) (p-value <0.01). Functional enrichment analysis and network analysis were conducted using the BiNGO version 2.3 [25], a Cytoscape [26] plug-in to identify biological processes overrepresented by target genes, resulted modulated by miRNAs detected in MVs (miRNAs co-expressed by MSCs and HLSCs, by cells and MVs or MV-enriched miRNAs). In the Gene Ontology (GO) tree, enriched Gene Ontology biological processes are represented by connected nodes with an associated statistical significance (P values derived from a hypergeometric hypothesis test in BiNGO). Biological functions showing p-value less than 0.05 were considered as significantly enriched.

## Supporting Information

Table S1 Fold change analysis of miRNAs coexpressed by MSCs and HLSCs. Normalized expression level of miRNAs from MSCs and HLSCs that clustered in the same expression groups are reported. The relative expression of miRNAs between MSCs and HLSCs was defined as fold change evaluated as 2-deltaCt.
(0.22 MB DOC)

Table S2 GO biological functions of targets of overexpressed miRNAs by cells and MVs. GO biological functions of predicted and validated targets of miRNAs overexpressed by cells and MVs, detected as % of cluster and total frequency of genes with a GO annotation. Only clusters with p value <0.05 are reported.
(0.16 MB DOC)

Table S3 GO biological functions of targets of miRNAs selectively present in MVs derived from HLSCs. GO biological functions of validated targets of miRNAs overexpressed by MVs derived from HLSCs, detected as % of cluster and total frequency of genes with a GO annotation. Only clusters with p value <0.05 are reported.
(0.20 MB DOC)

Table S4 GO biological functions of targets of miRNAs selectively present in MVs derived from MSCs. GO biological functions of validated targets of miRNAs overexpressed by MVs derived from MSCs, detected as % of cluster and total frequency of genes with a GO annotation. Only clusters with p value <0.05 are reported.
(0.22 MB DOC)

## References

[1] Schorey JS, Bhatnagar S (2008). Exosome function: from tumor immunology to pathogen biology.. Traffic.

[2] Skog J, Wurdinger T, van Rijn S, Meijer DH, Gainche L (2008). Glioblastoma microvesicles transport RNA and proteins that promote tumour growth and provide diagnostic biomarkers.. Nat Cell Biol.

[3] Ratajczak J, Miekus K, Kucia M, Zhang J, Reca R (2006). Embryonic stem cell-derived microvesicles reprogram hematopoietic progenitors: evidence for horizontal transfer of mRNA and protein delivery.. Leukemia.

[4] Deregibus MC, Cantaluppi V, Calogero R, Lo Iacono M, Tetta C (2007). Endothelial progenitor cell derived microvesicles activate an angiogenic program in endothelial cells by a horizontal transfer of mRNA.. Blood.

[5] Quesenberry PJ, Aliotta JM (2008). The paradoxical dynamism of marrow stem cells: considerations of stem cells, niches, and microvesicles.. Stem Cell Rev.

[6] Aliotta JM, Sanchez-Guijo FM, Dooner GJ, Johnson KW, Dooner MS (2007). Alteration of marrow cell gene expression, protein production, and engraftment into lung by lung-derived microvesicles: a novel mechanism for phenotype modulation.. Stem Cells.

[7] Deregibus MC, Tetta C, Camussi G (2010). The dynamic stem cell microenvironment is orchestrated by microvesicle-mediated transfer of genetic information.. Histol Histopathol.

[8] Bruno S, Grange C, Deregibus MC, Calogero RA, Saviozzi S (2009). Mesenchymal stem cell-derived microvesicles protect against acute tubular injury.. J Am Soc Nephrol.

[9] Herrera MB, Bruno S, Buttiglieri S, Tetta C, Gatti S (2006). Isolation and characterization of a stem cell population from adult human liver.. Stem Cells.

[10] Herrera MB, Fonsato V, Gatti S, Deregibus MC, Sordi A (2009). Human liver stem cell-derived microvesicles accelerate hepatic regeneration in hepatectomized rats.. J Cell Mol Med.

[11] Yuan A, Farber EL, Rapoport AL, Tejada D, Deniskin R (2009). Transfer of microRNAs by embryonic stem cell microvesicles.. PLoS One.

[12] Bartel DP (2004). MicroRNAs: genomics, biogenesis, mechanism, and function.. Cell.

[13] Lagos-Quintana M, Rauhut R, Lendeckel W, Tuschl T (2001). Identification of novel genes coding for small expressed RNAs.. Science.

[14] Lau NC, Lim LP, Weinstein EG, Bartel DP (2001). An abundant class of tiny RNAs with probable regulatory roles in Caenorhabditis elegans.. Science.

[15] Valadi H, Ekstrom K, Bossios A, Sjostrand M, Lee JJ (2007). Exosome-mediated transfer of mRNAs and microRNAs is a novel mechanism of genetic exchange between cells.. Nat Cell Biol.

[16] Hunter MP, Ismail N, Zhang X, Aguda BD, Lee EJ (2008). Detection of microRNA expression in human peripheral blood microvesicles.. PLoS One.

[17] Dahiya N, Sherman-Baust CA, Wang TL, Davidson B, Shih Ie M (2008). MicroRNA expression and identification of putative miRNA targets in ovarian cancer.. PLoS One.

[18] Chen TS, Lai RC, Lee MM, Choo AB, Lee CN (2010). Mesenchymal stem cell secretes microparticles enriched in pre-microRNAs.. Nucleic Acids Res.

[19] Anderson P, Kedersha N (2006). RNA granules.. J Cell Biol.

[20] Thomas MG, Martinez Tosar LJ, Loschi M, Pasquini JM, Correale J (2005). Staufen recruitment into stress granules does not affect early mRNA transport in oligodendrocytes.. Mol Biol Cell.

[21] Hock J, Meister G (2008). The Argonaute protein family.. Genome Biol.

[22] Doublier S, Musante L, Lupia E, Candiano G, Spatola T (2005). Direct effect of plasma permeability factors from patients with idiopatic FSGS on nephrin and podocin expression in human podocytes.. Int J Mol Med.

[23] Mestdagh P, Van Vlierberghe P, De Weer A, Muth D, Westermann F (2009). A novel and universal method for microRNA RT-qPCR data normalization.. Genome Biol.

[24] Bae S, Ahn JH, Park CW, Son HK, Kim KS (2009). Gene and microRNA expression signatures of human mesenchymal stromal cells in comparison to fibroblasts.. Cell Tissue Res.

[25] Maere S, Heymans K, Kuiper M (2005). BiNGO: a Cytoscape plugin to assess overrepresentation of gene ontology categories in biological networks.. Bioinformatics.

[26] Shannon P, Markiel A, Ozier O, Baliga NS, Wang JT (2003). Cytoscape: a software environment for integrated models of biomolecular interaction networks.. Genome Res.

[27] Lee Y, Kim M, Han J, Yeom KH, Lee S (2004). MicroRNA genes are transcribed by RNA polymerase II.. EMBO J.

[28] Meng F, Henson R, Wehbe-Janek H, Ghoshal K, Jacob ST (2007). MicroRNA-21 regulates expression of the PTEN tumor suppressor gene in human hepatocellular cancer.. Gastroenterology.

[29] Park JK, Lee EJ, Esau C, Schmittgen TD (2009). Antisense inhibition of microRNA-21 or -221 arrests cell cycle, induces apoptosis, and sensitizes the effects of gemcitabine in pancreatic adenocarcinoma.. Pancreas.

[30] Ohlsson Teague EM, Van der Hoek KH, Van der Hoek MB, Perry N, Wagaarachchi P (2009). MicroRNA-regulated pathways associated with endometriosis.. Mol Endocrinol.

[31] Ji Q, Hao X, Zhang M, Tang W, Yang M (2009). MicroRNA miR-34 inhibits human pancreatic cancer tumor-initiating cells.. PLoS One.

[32] Zhu W, Shan X, Wang T, Shu Y, Liu P (2010). miR-181b modulates multidrug resistance by targeting BCL2 in human cancer cell lines.. Int J Cancer.

[33] Xia L, Zhang D, Du R, Pan Y, Zhao L (2008). miR-15b and miR-16 modulate multidrug resistance by targeting BCL2 in human gastric cancer cells.. Int J Cancer.

[34] Zhou X, Ren Y, Moore L, Mei M, You Y (2010). Downregulation of miR-21 inhibits EGFR pathway and suppresses the growth of human glioblastoma cells independent of PTEN status.. Lab Invest.

[35] Ratajczak J, Wysoczynski M, Hayek F, Janowska-Wieczorek A, Ratajczak MZ (2006). Membrane-derived microvesicles: important and underappreciated mediators of cell-to-cell communication.. Leukemia.

[36] Heijnen HF, Schiel AE, Fijnheer R, Geuze HJ, Sixma JJ (1999). Activated platelets release two types of membrane vesicles: microvesicles by surface shedding and exosomes derived from exocytosis of multivesicular bodies and alpha-granules.. Blood.

[37] Rozmyslowicz T, Majka M, Kijowski J, Murphy SL, Conover DO (2003). Platelet- and megakaryocyte-derived microparticles transfer CXCR4 receptor to CXCR4-null cells and make them susceptible to infection by X4-HIV.. AIDS.

[38] Cocucci E, Racchetti G, Podini P, Meldolesi J (2007). Enlargeosome traffic: exocytosis triggered by various signals is followed by endocytosis, membrane shedding or both.. Traffic.

[39] Diamant M, Tushuizen ME, Sturk A, Nieuwland R (2004). Cellular microparticles: new players in the field of vascular disease?. Eur J Clin Invest.

[40] George JN, Thoi LL, McManus LM, Reimann TA (1982). Isolation of human platelet membrane microparticles from plasma and serum.. Blood.

[41] Martinez MC, Tesse A, Zobairi F, Andriantsitohaina R (2005). Shed membrane microparticles from circulating and vascular cells in regulating vascular function.. Am J Physiol Heart Circ Physiol.

[42] Morel O, Toti F, Hugel B, Freyssinet JM (2004). Cellular microparticles: a disseminated storage pool of bioactive vascular effectors.. Curr Opin Hematol.

[43] Dooner MS, Aliotta JM, Pimentel J, Dooner GJ, Abedi M (2008). Conversion potential of marrow cells into lung cells fluctuates with cytokine-induced cell cycle.. Stem Cells Dev.

[44] Gibbings DJ, Ciaudo C, Erhardt M, Voinnet O (2009). Multivesicular bodies associate with components of miRNA effector complexes and modulate miRNA activity.. Nat Cell Biol.

[45] Anderson P, Kedersha N (2009). Stress granules.. Curr Biol.

[46] Ivanov PA, Chudinova EM, Nadezhdina ES (2003). Disruption of microtubules inhibits cytoplasmic ribonucleoprotein stress granule formation.. Exp Cell Res.

[47] Moser JJ, Fritzler MJ (2009). Cytoplasmic ribonucleoprotein (RNP) bodies and their relationship to GW/P bodies.. Int J Biochem Cell Biol.

[48] Singh SR, Hou SX (2008). Immunohistological techniques for studying the Drosophila male germline stem cell.. Methods Mol Biol.

[49] Ballas N, Grunseich C, Lu DD, Speh JC, Mandel G (2005). REST and its corepressors mediate plasticity of neuronal gene chromatin throughout neurogenesis.. Cell.

[50] Stadler BM, Ruohola-Baker H (2008). Small RNAs: keeping stem cells in line.. Cell.

[51] Tay YM, Tam WL, Ang YS, Gaughwin PM, Yang H (2008). MicroRNA-134 modulates the differentiation of mouse embryonic stem cells, where it causes post-transcriptional attenuation of Nanog and LRH1.. Stem Cells.

[52] Valtieri M, Sorrentino A (2008). The mesenchymal stromal cell contribution to homeostasis.. J Cell Physiol.

[53] Greco SJ, Rameshwar P (2007). MicroRNAs regulate synthesis of the neurotransmitter substance P in human mesenchymal stem cell-derived neuronal cells.. Proc Natl Acad Sci U S A.

[54] Lakshmipathy U, Hart RP (2008). Concise review: MicroRNA expression in multipotent mesenchymal stromal cells.. Stem Cells.

[55] Masaki S, Ohtsuka R, Abe Y, Umemura T (2008). [Expression analysis of microRNAs in erythropoiesis].. Rinsho Byori.

[56] Dore LC, Amigo JD, Dos Santos CO, Zhang Z, Gai X (2008). A GATA-1-regulated microRNA locus essential for erythropoiesis.. Proc Natl Acad Sci U S A.

[57] Williams AE, Moschos SA, Perry MM, Barnes PJ, Lindsay MA (2007). Maternally imprinted microRNAs are differentially expressed during mouse and human lung development.. Dev Dyn.

[58] Bandres E, Bitarte N, Arias F, Agorreta J, Fortes P (2009). microRNA-451 regulates macrophage migration inhibitory factor production and proliferation of gastrointestinal cancer cells.. Clin Cancer Res.

[59] Fazi F, Rosa A, Fatica A, Gelmetti V, De Marchis ML (2005). A minicircuitry comprised of microRNA-223 and transcription factors NFI-A and C/EBPalpha regulates human granulopoiesis.. Cell.

[60] Johnnidis JB, Harris MH, Wheeler RT, Stehling-Sun S, Lam MH (2008). Regulation of progenitor cell proliferation and granulocyte function by microRNA-223.. Nature.

[61] Wong QW, Lung RW, Law PT, Lai PB, Chan KY (2008). MicroRNA-223 is commonly repressed in hepatocellular carcinoma and potentiates expression of Stathmin1.. Gastroenterology.

[62] Zernecke A, Bidzhekov K, Noels H, Shagdarsuren E, Gan L (2009). Delivery of microRNA-126 by apoptotic bodies induces CXCL12-dependent vascular protection.. Sci Signal.

[63] Koh W, Sheng CT, Tan B, Lee QY, Kuznetsov V (2010). Analysis of deep sequencing microRNA expression profile from human embryonic stem cells derived mesenchymal stem cells reveals possible role of let-7 microRNA family in downstream targeting of hepatic nuclear factor 4 alpha.. BMC Genomics.

[64] Wysoczynski M, Ratajczak MZ (2009). Lung cancer secreted microvesicles: underappreciated modulators of microenvironment in expanding tumors.. Int J Cancer.

[65] Kedersha N, Anderson P (2007). Mammalian stress granules and processing bodies.. Methods Enzymol.

[66] Sterpone L, Collino F, Camussi GC, L, Arabnia H (forthcoming) Analysis and Clustering of MicroRNA Array: A New Efficient And Reliable Computational Method.. Software Tools and Algorithms for Biological Systems (book series, Advances in Experimental Medicine and Biology, AEMB).

[67] Schmittgen TD, Lee EJ, Jiang J, Sarkar A, Yang L (2008). Real-time PCR quantification of precursor and mature microRNA.. Methods.

