# Supplementary material for: Microvesicles Derived from Adult Human Bone Marrow and Tissue Specific Mesenchymal Stem Cells Shuttle Selected Pattern of miRNAs
Source: PLoS One. 2010 Jul 27;5(7):e11803. doi: 10.1371/journal.pone.0011803 (PMC2910725; doi:10.1371/journal.pone.0011803)
Supplement: Table S1 — Fold change analysis of miRNAs coexpressed by MSCs and HLSCs. Normalized expression level of miRNAs from MSCs and HLSCs that clustered in the same expression groups are reported. The relative expression of miRNAs between MSCs and HLSCs was defined as fold change evaluated as 2-deltaCt. (0.22 MB DOC) [file pone.0011803.s001.doc]

**Table S1:** Fold change analysis of miRNAs coexpressed by MSCs and HLSCs.

| **Cluster** | | **miRNAs** | **MSC normalized expression±SD** | **HLSC normalized expression±SD** | **Fold change (MSC vs HLSC)** | **Fold change (HLSC vs MSC)** |
| --- | --- | --- | --- | --- | --- | --- |
| **< 0** | **hsa-miR-24** | | -6.37 ± 0.11 | -5.49 ± 0.35 | 0.54 | 1.84 |
|  | **hsa-miR-222** | | -7.13 ± 0.30 | -5.39 ± 0.55 | 0.3 | **3.35** |
|  | **hsa-miR-99a** | | -6.31 ± 0.10 | -5.21 ± 0.25 | 0.47 | **2.14** |
|  | **hsa-miR-125b** | | -7.52 ± 0.28 | -5.02 ± 0.01 | 0.18 | **5.66** |
|  | **hsa-miR-100** | | -6.19 ± 0.01 | -4.99 ± 0.01 | 0.44 | **2.29** |
|  | **hsa-miR-31** | | -5.27 ± 0.14 | -4.73 ± 0.57 | 0.68 | 1.46 |
|  | **hsa-miR-19b** | | -3.98 ± 0.14 | -4.44 ± 0.25 | 1.37 | 0.73 |
|  | **hsa-miR-16** | | -5.10 ± 0.05 | -4.19 ± 0.23 | 0.53 | 1.87 |
|  | **hsa-miR-594** | | -5.55 ± 0.15 | -4.01 ± 0.32 | 0.34 | **2.92** |
|  | **hsa-miR-125a** | | -5.10 ± 0.17 | -3.84 ± 0.30 | 0.42 | **2.39** |
|  | **hsa-miR-21** | | -4.74 ± 0.15 | -3.79 ± 0.31 | 0.52 | 1.93 |
|  | **hsa-miR-26a** | | -4.57 ± 0.01 | -3.35 ± 0.18 | 0.43 | **2.33** |
|  | **hsa-miR-221** | | -4.36 ± 0.21 | -3.09 ± 0.12 | 0.41 | **2.42** |
|  | **hsa-miR-565** | | -5.05 ± 1.18 | -2.88 ± 0.90 | 0.22 | **4.51** |
|  | **hsa-miR-92** | | -2.10 ± 0.34 | -2.88 ± 1.20 | 1.71 | 0.58 |
|  | **hsa-miR-99b** | | -2.66 ± 0.05 | -2.59 ± 0.47 | 0.96 | 1.04 |
|  | **hsa-miR-27a** | | -3.32 ± 0.09 | -2.54 ± 0.51 | 0.58 | 1.71 |
|  | **hsa-let-7b** | | -3.93 ± 0.34 | -2.51 ± 0.69 | 0.37 | **2.68** |
|  | **hsa-miR-214** | | -3.71 ± 0.50 | -2.44 ± 0.29 | 0.41 | **2.42** |
|  | **hsa-miR-484** | | -2.00 ± 0.32 | -2.37 ± 0.48 | 1.29 | 0.77 |
|  | **hsa-miR-20a** | | -1.28 ± 0.04 | -2.22 ± 0.09 | 1.92 | 0.52 |
|  | **hsa-miR-15b** | | -1.57 ± 0.01 | -1.94 ± 0.10 | 1.29 | 0.77 |
|  | **hsa-miR-29a** | | -2.94 ± 0.00 | -1.89 ± 0.12 | 0.48 | **2.08** |
|  | **hsa-miR-191** | | -2.29 ± 0.03 | -1.82 ± 0.31 | 0.72 | 1.39 |
|  | **hsa-miR-365** | | -3.51 ± 0.03 | -1.72 ± 0.67 | 0.29 | **3.47** |
|  | **hsa-miR-10a** | | -0.43 ± 0.24 | -1.67 ± 0.40 | **2.36** | 0.42 |
|  | **hsa-miR-30c** | | -2.35 ± 0.03 | -1.63 ± 0.41 | 0.61 | 1.65 |
|  | **hsa-miR-199a** | | -2.98 ± 0.78 | -1.56 ± 1.30 | 0.37 | **2.68** |
|  | **hsa-miR-93** | | -0.69 ± 0.10 | -1.49 ± 0.15 | 1.75 | 0.57 |
|  | **hsa-miR-127** | | -2.42 ± 0.10 | -1.30 ± 0.42 | 0.46 | **2.18** |
|  | **hsa-miR-19a** | | -0.10 ± 0.07 | -1.16 ± 0.20 | **2.09** | 0.48 |
|  | **hsa-miR-186** | | -0.84 ± 0.02 | -1.16 ± 0.20 | 1.25 | 0.8 |
|  | **hsa-miR-331** | | -1.50 ± 0.32 | -1.07 ± 0.58 | 0.74 | 1.35 |
|  | **hsa-miR-30b** | | -1.90 ± 0.17 | -0.90 ± 0.15 | 0.5 | 2 |
|  | **hsa-miR-342** | | -0.45 ± 0.03 | -0.77 ± 0.39 | 1.25 | 0.8 |
|  | **hsa-let-7a** | | -2.44 ± 0.43 | -0.74 ± 1.02 | 0.31 | **3.26** |
|  | **hsa-miR-151** | | -0.67 ± 0.12 | -0.71 ± 0.49 | 1.03 | 0.97 |
|  | **hsa-miR-106b** | | -0.14 ± 0.04 | -0.67 ± 0.23 | 1.45 | 0.69 |
|  | **hsa-miR-324-3p** | | -0.75 ± 0.10 | -0.54 ± 0.40 | 0.86 | 1.16 |
|  | **hsa-miR-197** | | -1.06 ± 0.26 | -0.51 ± 0.02 | 0.69 | 1.46 |
|  | **hsa-miR-26b** | | -1.54 ± 0.00 | -0.51 ± 0.05 | 0.49 | **2.04** |
|  | **hsa-miR-320** | | -0.90 ± 0.21 | -0.41 ± 0.39 | 0.71 | 1.41 |
|  | **hsa-miR-210** | | -2.93 ± 0.12 | -0.35 ± 0.44 | 0.17 | **5.98** |
|  | **hsa-miR-103** | | -1.30 ± 0.25 | -0.32 ± 0.40 | 0.51 | 1.98 |
|  | **hsa-miR-140** | | -2.26 ± 0.08 | -0.27 ± 0.22 | 0.25 | **3.97** |
|  | **hsa-miR-22** | | -0.42 ± 0.25 | -0.15 ± 0.12 | 0.83 | 1.2 |
| **> 0** | **hsa-miR-130a** | | 1.12 ± 0.27 | 0.54 ± 0.43 | 1.49 | 0.67 |
|  | **hsa-miR-25** | | 0.90 ± 0.04 | 0.58 ± 0.44 | 1.25 | 0.8 |
|  | **hsa-miR-660** | | 0.41 ± 0.11 | 1.09 ± 0.15 | 0.62 | 1.6 |
|  | **hsa-miR-487b** | | 1.55 ± 0.20 | 1.23 ± 0.12 | 1.25 | 0.8 |
|  | **hsa-miR-30e-3p** | | 0.49 ± 0.01 | 1.24 ± 0.03 | 0.6 | 1.68 |
|  | **hsa-miR-411** | | 0.25 ± 0.28 | 1.29 ± 0.51 | 0.49 | **2.06** |
|  | **hsa-miR-328** | | 0.30 ± 0.03 | 1.31 ± 0.04 | 0.5 | **2.02** |
|  | **hsa-miR-361** | | 1.27 ± 0.84 | 1.33 ± 0.10 | 0.96 | 1.05 |
|  | **hsa-miR-425-5p** | | 0.97 ± 0.66 | 1.45 ± 0.12 | 0.72 | 1.39 |
|  | **hsa-let-7f** | | 1.27 ± 0.25 | 1.45 ± 0.68 | 0.88 | 1.14 |
|  | **hsa-miR-30a-3p** | | 0.70 ± 0.13 | 1.54 ± 0.07 | 0.56 | 1.78 |
|  | **hsa-miR-23a** | | 1.33 ± 0.21 | 1.58 ± 0.25 | 0.84 | 1.19 |
|  | **hsa-miR-485-3p** | | 0.69 ± 0.01 | 1.64 ± 0.18 | 0.52 | 1.93 |
|  | **hsa-miR-301** | | 1.42 ± 0.12 | 1.66 ± 0.46 | 0.85 | 1.18 |
|  | **hsa-miR-532** | | 1.91 ± 0.18 | 1.73 ± 0.34 | 1.14 | 0.88 |
|  | **hsa-miR-146b** | | 1.53 ± 0.21 | 1.75 ± 0.53 | 0.86 | 1.16 |
|  | **hsa-miR-324-5p** | | 1.53 ± 0.56 | 1.83 ± 0.86 | 0.82 | 1.23 |
|  | **hsa-miR-423** | | 1.56 ± 0.08 | 1.86 ± 0.03 | 0.81 | 1.24 |
|  | **hsa-miR-432** | | 1.44 ± 0.18 | 1.88 ± 0.90 | 0.74 | 1.36 |
|  | **hsa-miR-376a** | | 1.67 ± 0.23 | 1.91 ± 0.12 | 0.85 | 1.18 |
|  | **hsa-miR-345** | | 2.81 ± 0.66 | 2.01 ± 0.25 | 1.74 | 0.57 |
|  | **hsa-miR-296** | | 1.22 ± 0.08 | 2.17 ± 0.21 | 0.52 | 1.93 |
|  | **hsa-let-7e** | | 1.35 ± 0.68 | 2.24 ± 0.76 | 0.54 | 1.85 |
|  | **hsa-miR-299-5p** | | 2.03 ± 0.10 | 2.24± 0.86 | 0.86 | 1.16 |
|  | **hsa-miR-32** | | 3.34 ± 0.70 | 2.28 ± 0.49 | **2.08** | 0.48 |
|  | **hsa-miR-550** | | 0.95 ± 0.01 | 2.32 ± 0.18 | 0.39 | **2.59** |
|  | **hsa-miR-218** | | 1.29 ± 0.38 | 2.40 ± 0.98 | 0.46 | **2.16** |
|  | **hsa-miR-199a** | | 2.77 ± 0.73 | 2.53 ± 1.25 | 1.18 | 0.85 |
|  | **hsa-miR-485-5p** | | 1.75 ± 0.44 | 2.54 ± 0.64 | 0.58 | 1.74 |
|  | **hsa-miR-98** | | 1.84 ± 0.19 | 2.84 ± 0.79 | 0.5 | 1.99 |
|  | **hsa-let-7d** | | 2.87 ±0.27 | 2.84 ± 0.19 | 1.02 | 0.98 |
|  | **hsa-miR-199b** | | 1.35 ± 0.53 | 2.89 ± 0.82 | 0.34 | **2.92** |
|  | **hsa-miR-148b** | | 1.70 ± 1.03 | 2.92 ± 1.57 | 0.43 | **2.33** |
|  | **hsa-miR-213** | | 3.87 ± 0.44 | 2.97 ± 0.03 | 1.87 | 0.54 |
|  | **hsa-let-7c** | | 1.29 ± 0.16 | 3.11 ± 0.66 | 0.28 | **3.53** |
|  | **hsa-miR-34a** | | 2.80 ± 0.11 | 3.14 ± 0.37 | 0.79 | 1.27 |
|  | **hsa-miR-452** | | 2.32 ± 0.16 | 3.25 ± 0.37 | 0.53 | 1.9 |
|  | **hsa-miR-362** | | 2.91 ± 0.40 | 3.31 ± 0.72 | 0.76 | 1.32 |
|  | **hsa-miR-192** | | 3.34 ± 0.32 | 3.31 ± 0.73 | 1.02 | 0.98 |
|  | **hsa-miR-491** | | 2.31 ± 0.29 | 3.58 ± 1.07 | 0.42 | **2.4** |
|  | **hsa-miR-500** | | 2.27 ± 0.10 | 3.66 ± 0.34 | 0.38 | **2.62** |
|  | **hsa-miR-410** | | 3.33 ± 0.28 | 3.67 ± 0.43 | 0.79 | 1.27 |
|  | **hsa-miR-193b** | | 1.63 ± 0.39 | 3.68 ± 0.94 | 0.24 | **4.14** |
|  | **hsa-miR-29c** | | 2.97 ± 0.68 | 3.79 ± 0.06 | 0.57 | 1.77 |
|  | **hsa-miR-379** | | 3.03 ± 0.22 | 3.85 ± 0.38 | 0.57 | 1.76 |
|  | **hsa-miR-433** | | 2.32 ± 0.28 | 3.85 ± 1.01 | 0.35 | **2.89** |
|  | **hsa-miR-181c** | | 3.07 ± 0.42 | 3.88 ± 0.23 | 0.57 | 1.75 |
|  | **hsa-miR-330** | | 4.84 ± 0.15 | 4.22 ± 0.29 | 1.53 | 0.65 |
|  | **hsa-miR-629** | | 4.66 ± 0.05 | 4.31 ± 0.43 | 1.27 | 0.78 |
|  | **hsa-miR-425** | | 2.49 ± 0.26 | 4.36 ± 0.06 | 0.27 | **3.67** |
|  | **hsa-miR-452** | | 3.68 ± 0.51 | 4.39 ± 0.62 | 0.61 | 1.64 |
|  | **hsa-miR-502** | | 4.51 ± 0.05 | 4.79 ± 0.18 | 0.82 | 1.22 |

Normalized expression level of miRNAs from MSCs and HLSCs that clustered in the same expression groups are reported. The relative expression of miRNAs between MSCs and HLSCs was defined as fold change evaluated as 2-ΔCt.
