# Supplementary material for: Microvesicles Derived from Adult Human Bone Marrow and Tissue Specific Mesenchymal Stem Cells Shuttle Selected Pattern of miRNAs
Source: PLoS One. 2010 Jul 27;5(7):e11803. doi: 10.1371/journal.pone.0011803 (PMC2910725; doi:10.1371/journal.pone.0011803)
Supplement: Table S2 — GO biological functions of targets of overexpressed miRNAs by cells and MVs. GO biological functions of predicted and validated targets of miRNAs overexpressed by cells and MVs, detected as % of cluster and total frequency of genes with a GO annotation. Only clusters with p value <0.05 are reported. (0.16 MB DOC) [file pone.0011803.s002.doc]

**Table S2: GO biological functions of targets of overexpressed miRNAs by cells and MVs.**

| **GO-ID** | **GO biological function** | **p-value** | **corr p-value** | **cluster freq.** | **total freq.** |
| --- | --- | --- | --- | --- | --- |
| 48523 | negative regulation of cellular process | 2.7071E-24 | 8.557E-21 | 224/1513 14.8% | 1081/14444 7.4% |
| 48519 | negative regulation of biological process | 1.3864E-23 | 4.3824E-20 | 232/1513 15.3% | 1149/14444 7.9% |
| 50793 | regulation of developmental process | 3.5136E-21 | 1.1106E-17 | 171/1513 11.3% | 777/14444 5.3% |
| 48518 | positive regulation of biological process | 1.7468E-16 | 5.5216E-13 | 208/1513 13.7% | 1122/14444 7.7% |
| 51242 | positive regulation of cellular process | 1.9609E-16 | 6.1983E-13 | 193/1513 12.7% | 1015/14444 7.0% |
| 51094 | positive regulation of developmental process | 7.5067E-16 | 2.3729E-12 | 92/1513 6.0% | 356/14444 2.4% |
| 43067 | regulation of programmed cell death | 1.317E-15 | 4.163E-12 | 117/1513 7.7% | 513/14444 3.5% |
| 32502 | developmental process | 1.5039E-15 | 4.7539E-12 | 408/1513 26.9% | 2721/14444 18.8% |
| 42981 | regulation of apoptosis | 3.1988E-15 | 1.0111E-11 | 115/1513 7.6% | 506/14444 3.5% |
| 43412 | biopolymer modification | 5.9402E-15 | 1.8777E-11 | 236/1513 15.5% | 1367/14444 9.4% |
| 6464 | protein modification process | 5.87E-14 | 1.8555E-10 | 225/1513 14.8% | 1310/14444 9.0% |
| 48731 | system development | 2.0202E-13 | 6.3858E-10 | 250/1513 16.5% | 1517/14444 10.5% |
| 48856 | anatomical structure development | 1.5164E-12 | 4.7934E-09 | 273/1513 18.0% | 1725/14444 11.9% |
| 9892 | negative regulation of metabolic process | 3.6693E-12 | 1.1599E-08 | 96/1513 6.3% | 431/14444 2.9% |
| 43687 | post-translational protein modification | 4.2317E-12 | 1.3376E-08 | 176/1513 11.6% | 991/14444 6.8% |
| 48513 | organ development | 4.2907E-12 | 1.3563E-08 | 187/1513 12.3% | 1073/14444 7.4% |
| 31324 | negative regulation of cellular metabolic process | 4.5369E-12 | 1.4341E-08 | 95/1513 6.2% | 426/14444 2.9% |
| 65009 | regulation of molecular function | 2.4477E-11 | 7.7372E-08 | 125/1513 8.2% | 641/14444 4.4% |
| 51093 | negative regulation of developmental process | 8.1496E-11 | 2.5761E-07 | 75/1513 4.9% | 318/14444 2.2% |
| 42127 | regulation of cell proliferation | 1.0734E-10 | 3.3931E-07 | 100/1513 6.6% | 482/14444 3.3% |
| 43065 | positive regulation of apoptosis | 1.3701E-10 | 4.3308E-07 | 63/1513 4.1% | 248/14444 1.7% |
| 43068 | positive regulation of programmed cell death | 2.3882E-10 | 7.5492E-07 | 63/1513 4.1% | 251/14444 1.7% |
| 6917 | induction of apoptosis | 4.7226E-10 | 1.4928E-06 | 53/1513 3.5% | 196/14444 1.3% |
| 45859 | regulation of protein kinase activity | 4.8345E-10 | 1.5282E-06 | 59/1513 3.8% | 231/14444 1.5% |
| 7275 | multicellular organismal development | 5.2977E-10 | 1.6746E-06 | 308/1513 20.3% | 2102/14444 14.5% |
| 45934 | negative regulation of nucleobase. nucleoside. nucleotide and nucleic acid metabolic process | 5.3754E-10 | 1.6992E-06 | 72/1513 4.7% | 311/14444 2.1% |
| 12502 | induction of programmed cell death | 5.8161E-10 | 1.8385E-06 | 53/1513 3.5% | 197/14444 1.3% |
| 8632 | apoptotic program | 1.0001E-09 | 3.1613E-06 | 33/1513 2.1% | 93/14444 0.6% |
| 43549 | regulation of kinase activity | 1.2217E-09 | 3.8618E-06 | 59/1513 3.8% | 236/14444 1.6% |
| 50790 | regulation of catalytic activity | 1.3056E-09 | 4.1271E-06 | 111/1513 7.3% | 579/14444 4.0% |
| 7242 | intracellular signaling cascade | 1.9308E-09 | 6.1031E-06 | 189/1513 12.4% | 1164/14444 8.0% |
| 10605 | negative regulation of macromolecule metabolic process | 2.451E-09 | 7.7477E-06 | 82/1513 5.4% | 386/14444 2.6% |
| 51338 | regulation of transferase activity | 2.9806E-09 | 9.4218E-06 | 59/1513 3.8% | 241/14444 1.6% |
| 48869 | cellular developmental process | 3.0493E-09 | 9.6389E-06 | 178/1513 11.7% | 1085/14444 7.5% |
| 10629 | negative regulation of gene expression | 3.9678E-09 | 0.000012542 | 66/1513 4.3% | 286/14444 1.9% |
| 9890 | negative regulation of biosynthetic process | 4.181E-09 | 0.000013216 | 73/1513 4.8% | 331/14444 2.2% |
| 6468 | protein amino acid phosphorylation | 4.9948E-09 | 0.000015789 | 113/1513 7.4% | 606/14444 4.1% |
| 16481 | negative regulation of transcription | 5.6795E-09 | 0.000017953 | 65/1513 4.2% | 282/14444 1.9% |
| 30154 | cell differentiation | 7.2005E-09 | 0.000022761 | 166/1513 10.9% | 1004/14444 6.9% |
| 43069 | negative regulation of programmed cell death | 8.9397E-09 | 0.000028258 | 54/1513 3.5% | 217/14444 1.5% |
| 10558 | negative regulation of macromolecule biosynthetic process | 1.0462E-08 | 0.00003307 | 70/1513 4.6% | 318/14444 2.2% |
| 43066 | negative regulation of apoptosis | 1.5355E-08 | 0.000048538 | 53/1513 3.5% | 214/14444 1.4% |
| 7167 | enzyme linked receptor protein signaling pathway | 2.2177E-08 | 0.000070102 | 59/1513 3.8% | 253/14444 1.7% |
| 12501 | programmed cell death | 3.6781E-08 | 0.00011626 | 85/1513 5.6% | 428/14444 2.9% |
| 43283 | biopolymer metabolic process | 4.0417E-08 | 0.00012776 | 527/1513 34.8% | 4097/14444 28.3% |
| 51253 | negative regulation of RNA metabolic process | 4.5387E-08 | 0.00014347 | 50/1513 3.3% | 202/14444 1.3% |
| 6915 | apoptosis | 5.1317E-08 | 0.00016221 | 84/1513 5.5% | 424/14444 2.9% |
| 6796 | phosphate metabolic process | 5.3255E-08 | 0.00016834 | 146/1513 9.6% | 877/14444 6.0% |
| 6793 | phosphorus metabolic process | 5.3255E-08 | 0.00016834 | 146/1513 9.6% | 877/14444 6.0% |
| 16043 | cellular component organization and biogenesis | 5.4237E-08 | 0.00017144 | 248/1513 16.3% | 1689/14444 11.6% |
| 8219 | cell death | 7.3805E-08 | 0.0002333 | 92/1513 6.0% | 483/14444 3.3% |
| 16265 | death | 7.3805E-08 | 0.0002333 | 92/1513 6.0% | 483/14444 3.3% |
| 45892 | negative regulation of transcription. DNA-dependent | 7.7785E-08 | 0.00024588 | 49/1513 3.2% | 199/14444 1.3% |
| 16310 | phosphorylation | 2.9945E-07 | 0.00094656 | 122/1513 8.0% | 717/14444 4.9% |
| 45597 | positive regulation of cell differentiation | 3.0405E-07 | 0.00096109 | 25/1513 1.6% | 72/14444 0.4% |
| 79 | regulation of cyclin-dependent protein kinase activity | 3.1789E-07 | 0.001 | 20/1513 1.3% | 49/14444 0.3% |
| 74 | regulation of cell cycle | 3.4423E-07 | 0.001 | 62/1513 4.0% | 291/14444 2.0% |
| 10604 | positive regulation of macromolecule metabolic process | 3.7424E-07 | 0.001 | 79/1513 5.2% | 407/14444 2.8% |
| 6357 | regulation of transcription from RNA polymerase II promoter | 4.6368E-07 | 0.001 | 83/1513 5.4% | 437/14444 3.0% |
| 9893 | positive regulation of metabolic process | 5.116E-07 | 0.002 | 84/1513 5.5% | 445/14444 3.0% |
| 45786 | negative regulation of cell cycle | 5.4162E-07 | 0.002 | 39/1513 2.5% | 150/14444 1.0% |
| 19538 | protein metabolic process | 5.6397E-07 | 0.002 | 369/1513 24.3% | 2765/14444 19.1% |
| 7399 | nervous system development | 7.0804E-07 | 0.002 | 117/1513 7.7% | 690/14444 4.7% |
| 6950 | response to stress | 8.3403E-07 | 0.003 | 189/1513 12.4% | 1256/14444 8.6% |
| 31325 | positive regulation of cellular metabolic process | 8.7173E-07 | 0.003 | 82/1513 5.4% | 436/14444 3.0% |
| 45595 | regulation of cell differentiation | 1.1406E-06 | 0.004 | 43/1513 2.8% | 178/14444 1.2% |
| 43170 | macromolecule metabolic process | 1.1562E-06 | 0.004 | 644/1513 42.5% | 5261/14444 36.4% |
| 9887 | organ morphogenesis | 1.4339E-06 | 0.005 | 67/1513 4.4% | 336/14444 2.3% |
| 44238 | primary metabolic process | 1.7731E-06 | 0.006 | 756/1513 49.9% | 6322/14444 43.7% |
| 8151 | cellular process | 2.547E-06 | 0.008 | 1250/1513 82.6% | 11211/14444 77.6% |
| 44267 | cellular protein metabolic process | 2.6856E-06 | 0.008 | 361/1513 23.8% | 2734/14444 18.9% |
| 52548 | regulation of endopeptidase activity | 0.00000306 | 0.010 | 20/1513 1.3% | 55/14444 0.3% |
| 44260 | cellular macromolecule metabolic process | 3.8037E-06 | 0.01 | 369/1513 24.3% | 2813/14444 19.4% |
| 6916 | anti-apoptosis | 4.1388E-06 | 0.01 | 37/1513 2.4% | 149/14444 1.0% |
| 52547 | regulation of peptidase activity | 4.2945E-06 | 0.01 | 20/1513 1.3% | 56/14444 0.3% |
| 16044 | membrane organization and biogenesis | 6.3886E-06 | 0.02 | 55/1513 3.6% | 267/14444 1.8% |
| 43281 | regulation of caspase activity | 7.787E-06 | 0.02 | 19/1513 1.2% | 53/14444 0.3% |
| 33673 | negative regulation of kinase activity | 8.2203E-06 | 0.03 | 20/1513 1.3% | 58/14444 0.4% |
| 6469 | negative regulation of protein kinase activity | 8.2203E-06 | 0.03 | 20/1513 1.3% | 58/14444 0.4% |
| 43086 | negative regulation of catalytic activity | 1.0997E-05 | 0.03 | 39/1513 2.5% | 167/14444 1.1% |
| 8633 | activation of pro-apoptotic gene products | 1.1015E-05 | 0.03 | 11/1513 0.7% | 20/14444 0.1% |
| 65008 | regulation of biological quality | 1.2761E-05 | 0.04 | 138/1513 9.1% | 893/14444 6.1% |
| 16070 | RNA metabolic process | 1.2973E-05 | 0.04 | 131/1513 8.6% | 838/14444 5.8% |
| 10557 | positive regulation of macromolecule biosynthetic process | 1.2974E-05 | 0.04 | 66/1513 4.3% | 349/14444 2.4% |
| 43085 | positive regulation of catalytic activity | 1.3057E-05 | 0.04 | 63/1513 4.1% | 328/14444 2.2% |
| 50865 | regulation of cell activation | 0.00001414 | 0.04 | 27/1513 1.7% | 97/14444 0.6% |
| 51348 | negative regulation of transferase activity | 1.5187E-05 | 0.05 | 20/1513 1.3% | 60/14444 0.4% |

GO biological functions of predicted and validated targets of miRNAs overexpressed by cells and MVs, detected as % of cluster and total frequency of genes with a GO annotation. Only clusters with *p* value <0.05 are reported.
