# Supplementary material for: Microvesicles Derived from Adult Human Bone Marrow and Tissue Specific Mesenchymal Stem Cells Shuttle Selected Pattern of miRNAs
Source: PLoS One. 2010 Jul 27;5(7):e11803. doi: 10.1371/journal.pone.0011803 (PMC2910725; doi:10.1371/journal.pone.0011803)
Supplement: Table S3 — GO biological functions of targets of miRNAs selectively present in MVs derived from HLSCs. GO biological functions of validated targets of miRNAs overexpressed by MVs derived from HLSCs, detected as % of cluster and total frequency of genes with a GO annotation. Only clusters with p value <0.05 are reported. (0.20 MB DOC) [file pone.0011803.s003.doc]

**Table S3: GO biological functions of targets of miRNAs selectively present in MVs derived from HLSCs.**

| **GO-ID** | **Biological process** | **p-value** | **corr p-value** | **cluster freq.** | | | **tot freq.** |
| --- | --- | --- | --- | --- | --- | --- | --- |
| 31325 | positive regulation of cellular metabolic process | 1.69E-17 | 2.03E-14 | 23/75 | | | 438/14519 |
| 9893 | positive regulation of metabolic process | 2.65E-17 | 3.17E-14 | 23/75 | | | 447/14519 |
| 10604 | positive regulation of macromolecule metabolic process | 5.89E-17 | 7.05E-14 | 22/75 | | | 409/14519 |
| 9891 | positive regulation of biosynthetic process | 1.22E-16 | 1.47E-13 | 21/75 | | | 371/14519 |
| 45935 | positive regulation of nucleobase. nucleoside. nucleotide and nucleic acid metabolic process | 1.86E-16 | 2.23E-13 | 20/75 | | | 329/14519 |
| 10557 | positive regulation of macromolecule biosynthetic process | 6.50E-16 | 7.79E-13 | 20/75 | | | 351/14519 |
| 45941 | positive regulation of transcription | 9.06E-16 | 1.09E-12 | 19/75 | | | 308/14519 |
| 10628 | positive regulation of gene expression | 1.37E-15 | 1.64E-12 | 19/75 | | | 315/14519 |
| 51242 | positive regulation of cellular process | 5.13E-15 | 6.15E-12 | 29/75 | | | 1018/14519 |
| 48518 | positive regulation of biological process | 8.30E-15 | 9.95E-12 | 30/75 | | | 1124/14519 |
| 45893 | positive regulation of transcription. DNA-dependent | 1.06E-13 | 1.27E-10 | 16/75 | | | 247/14519 |
| 51254 | positive regulation of RNA metabolic process | 1.20E-13 | 1.44E-10 | 16/75 | | | 249/14519 |
| 6357 | regulation of transcription from RNA polymerase II promoter | 5.31E-13 | 6.36E-10 | 19/75 | | | 438/14519 |
| 48523 | negative regulation of cellular process | 1.08E-11 | 1.30E-08 | 26/75 | | | 1084/14519 |
| 45944 | positive regulation of transcription from RNA polymerase II promoter | 2.25E-11 | 2.70E-08 | 12/75 | | | 156/14519 |
| 48519 | negative regulation of biological process | 4.23E-11 | 5.06E-08 | 26/75 | | | 1152/14519 |
| 50793 | regulation of developmental process | 2.21E-10 | 2.65E-07 | 21/75 | | | 780/14519 |
| 10468 | regulation of gene expression | 2.63E-10 | 3.15E-07 | 38/75 | | | 2680/14519 |
| 32502 | developmental process | 4.35E-10 | 5.21E-07 | 38/75 | | | 2725/14519 |
| 7275 | multicellular organismal development | 6.40E-10 | 7.67E-07 | 33/75 | | | 2103/14519 |
| 51244 | regulation of cellular process | 6.47E-10 | 7.75E-07 | 60/75 | | | 6565/14519 |
| 2376 | immune system process | 1.44E-09 | 1.72E-06 | 20/75 | | | 777/14519 |
| 19222 | regulation of metabolic process | 1.63E-09 | 1.96E-06 | 39/75 | | | 2991/14519 |
| 60255 | regulation of macromolecule metabolic process | 1.91E-09 | 2.29E-06 | 38/75 | | | 2864/14519 |
| 48513 | organ development | 2.10E-09 | 2.52E-06 | 23/75 | | | 1071/14519 |
| 50791 | regulation of biological process | 2.41E-09 | 2.88E-06 | 60/75 | | | 6747/14519 |
| 30154 | cell differentiation | 3.34E-09 | 4.00E-06 | 22/75 | | | 1000/14519 |
| 46649 | lymphocyte activation | 4.15E-09 | 4.97E-06 | 9/75 | | | 108/14519 |
| 65007 | biological regulation | 6.67E-09 | 7.99E-06 | 61/75 | | | 7114/14519 |
| 1666 | response to hypoxia | 1.23E-08 | 1.47E-05 | 7/75 | | | 55/14519 |
| 48869 | cellular developmental process | 1.37E-08 | 1.64E-05 | 22/75 | | | 1080/14519 |
| 42110 | T cell activation | 1.40E-08 | 1.67E-05 | 7/75 | | | 56/14519 |
| 48731 | system development | 1.59E-08 | 1.91E-05 | 26/75 | | | 1517/14519 |
| 42127 | regulation of cell proliferation | 1.88E-08 | 2.25E-05 | 15/75 | | | 481/14519 |
| 45321 | leukocyte activation | 2.28E-08 | 2.73E-05 | 9/75 | | | 131/14519 |
| 45595 | regulation of cell differentiation | 2.76E-08 | 3.30E-05 | 10/75 | | | 180/14519 |
| 31324 | negative regulation of cellular metabolic process | 3.10E-08 | 3.72E-05 | 14/75 | | | 426/14519 |
| 9892 | negative regulation of metabolic process | 3.59E-08 | 4.30E-05 | 14/75 | | | 431/14519 |
| 31323 | regulation of cellular metabolic process | 5.00E-08 | 5.98E-05 |  | 36/75 |  | 2913/14519 |
| 48856 | anatomical structure development | 5.31E-08 | 6.36E-05 | 27/75 | | | 1727/14519 |
| 45934 | negative regulation of nucleobase. nucleoside. nucleotide and nucleic acid metabolic process | 5.90E-08 | 7.07E-05 | 12/75 | | | 311/14519 |
| 10556 | regulation of macromolecule biosynthetic process | 6.64E-08 | 7.95E-05 | 34/75 | | | 2659/14519 |
| 45892 | negative regulation of transcription. DNA-dependent | 7.13E-08 | 8.55E-05 | 10/75 | | | 199/14519 |
| 10605 | negative regulation of macromolecule metabolic process | 7.71E-08 | 9.24E-05 | 13/75 | | | 386/14519 |
| 51253 | negative regulation of RNA metabolic process | 8.21E-08 | 9.84E-05 | 10/75 | | | 202/14519 |
| 9889 | regulation of biosynthetic process | 8.66E-08 | 1.04E-04 | 34/75 | | | 2687/14519 |
| 1775 | cell activation | 1.44E-07 | 1.72E-04 | 9/75 | | | 162/14519 |
| 22415 | viral reproductive process | 1.60E-07 | 1.92E-04 | 6/75 | | | 48/14519 |
| 16481 | negative regulation of transcription | 1.99E-07 | 2.38E-04 | 11/75 | | | 282/14519 |
| 6355 | regulation of transcription. DNA-dependent | 1.99E-07 | 2.39E-04 | 31/75 | | | 2360/14519 |
| 10629 | negative regulation of gene expression | 2.29E-07 | 2.74E-04 | 11/75 | | | 286/14519 |
| 51252 | regulation of RNA metabolic process | 2.31E-07 | 2.76E-04 | 31/75 | | | 2375/14519 |
| 51094 | positive regulation of developmental process | 2.64E-07 | 3.16E-04 | 12/75 | | | 357/14519 |
| 122 | negative regulation of transcription from RNA polymerase II promoter | 4.28E-07 | 5.13E-04 | 8/75 | | | 134/14519 |
| 19219 | regulation of nucleobase. nucleoside. nucleotide and nucleic acid metabolic process | 4.39E-07 | 5.26E-04 | 32/75 | | | 2582/14519 |
| 31047 | gene silencing by RNA | 4.53E-07 | 5.43E-04 | 4/75 | | | 13/14519 |
| 65008 | regulation of biological quality | 4.56E-07 | 5.46E-04 | 18/75 | | | 893/14519 |
| 16032 | viral reproduction | 4.57E-07 | 5.47E-04 | 6/75 | | | 57/14519 |
| 45076 | regulation of interleukin-2 biosynthetic process | 6.32E-07 | 7.57E-04 | 4/75 | | | 14/14519 |
| 10558 | negative regulation of macromolecule biosynthetic process | 6.58E-07 | 7.88E-04 | 11/75 | | | 318/14519 |
| 32583 | regulation of gene-specific transcription | 7.95E-07 | 9.53E-04 | 5/75 | | | 34/14519 |
| 45449 | regulation of transcription | 8.62E-07 | 1.03E-03 | 31/75 | | | 2517/14519 |
| 9890 | negative regulation of biosynthetic process | 9.76E-07 | 1.17E-03 | 11/75 | | | 331/14519 |
| 32501 | multicellular organismal process | 1.06E-06 | 1.28E-03 | 36/75 | | | 3275/14519 |
| 30097 | hemopoiesis | 2.46E-06 | 2.95E-03 | 7/75 | | | 118/14519 |
| 44419 | interspecies interaction between organisms | 2.63E-06 | 3.15E-03 | 9/75 | | | 229/14519 |
| 31401 | positive regulation of protein modification process | 5.13E-06 | 6.15E-03 | 5/75 | | | 49/14519 |
| 48534 | hemopoietic or lymphoid organ development | 5.20E-06 | 6.22E-03 | 7/75 | | | 132/14519 |
| 2520 | immune system development | 6.64E-06 | 7.96E-03 | 7/75 | | | 137/14519 |
| 7420 | brain development | 6.97E-06 | 8.35E-03 | 7/75 | | | 138/14519 |
| 51869 | response to stimulus | 7.10E-06 | 8.51E-03 | 30/75 | | | 2626/14519 |
| 43193 | positive regulation of gene-specific transcription | 7.65E-06 | 9.17E-03 | 4/75 | | | 25/14519 |
| 2694 | regulation of leukocyte activation | 8.81E-06 | 1.06E-02 | 6/75 | | | 94/14519 |
| 50865 | regulation of cell activation | 1.06E-05 | 1.27E-02 | 6/75 | | | 97/14519 |
| 16458 | gene silencing | 1.22E-05 | 1.47E-02 | 4/75 | | | 28/14519 |
| 44237 | cellular metabolic process | 1.39E-05 | 1.67E-02 | 52/75 | | | 6494/14519 |
| 45086 | positive regulation of interleukin-2 biosynthetic process | 1.55E-05 | 1.85E-02 | 3/75 | | | 10/14519 |
| 8361 | regulation of cell size | 1.76E-05 | 2.11E-02 | 6/75 | | | 106/14519 |
| 7242 | intracellular signaling cascade | 1.99E-05 | 2.39E-02 | 18/75 | | | 1167/14519 |
| 7417 | central nervous system development | 2.16E-05 | 2.59E-02 | 8/75 | | | 227/14519 |
| 35195 | gene silencing by miRNA | 2.63E-05 | 3.15E-02 | 2/75 | | | 2/14519 |
| 45368 | positive regulation of interleukin-13 biosynthetic process | 2.63E-05 | 3.15E-02 | 2/75 | | | 2/14519 |
| 45366 | regulation of interleukin-13 biosynthetic process | 2.63E-05 | 3.15E-02 | 2/75 | | | 2/14519 |
| 10039 | response to iron ion | 2.63E-05 | 3.15E-02 | 2/75 | | | 2/14519 |
| 31050 | dsRNA fragmentation | 2.63E-05 | 3.15E-02 | 2/75 | | | 2/14519 |
| 30918 | gene silencing by miRNA. production of miRNAs | 2.63E-05 | 3.15E-02 | 2/75 | | | 2/14519 |
| 35270 | endocrine system development | 2.71E-05 | 3.24E-02 | 4/75 | | | 34/14519 |
| 50863 | regulation of T cell activation | 2.79E-05 | 3.34E-02 | 5/75 | | | 69/14519 |
| 8285 | negative regulation of cell proliferation | 3.04E-05 | 3.64E-02 | 8/75 | | | 238/14519 |
| 1934 | positive regulation of protein amino acid phosphorylation | 3.81E-05 | 4.57E-02 | 4/75 | | | 37/14519 |

GO biological functions of validated targets of miRNAs overexpressed by MVs derived from HLSCs, detected as % of cluster and total frequency of genes with a GO annotation. Only clusters with *p* value <0.05 are reported.
