# Supplementary material for: Microvesicles Derived from Adult Human Bone Marrow and Tissue Specific Mesenchymal Stem Cells Shuttle Selected Pattern of miRNAs
Source: PLoS One. 2010 Jul 27;5(7):e11803. doi: 10.1371/journal.pone.0011803 (PMC2910725; doi:10.1371/journal.pone.0011803)
Supplement: Table S4 — GO biological functions of targets of miRNAs selectively present in MVs derived from MSCs. GO biological functions of validated targets of miRNAs overexpressed by MVs derived from MSCs, detected as % of cluster and total frequency of genes with a GO annotation. Only clusters with p value <0.05 are reported. (0.22 MB DOC) [file pone.0011803.s004.doc]

**Table S4: GO biological functions of targets of miRNAs selectively present in MVs derived from MSCs.**

| **GO-ID** | **GO biological function** | **p-value** | **corr p-value** | **cluster freq.** | **total freq.** |
| --- | --- | --- | --- | --- | --- |
| 10604 | positive regulation of macromolecule metabolic process | 2.122E-12 | 1.3999E-09 | 15/42 35.7% | 409/14524 2.8% |
| 10557 | positive regulation of macromolecule biosynthetic process | 5.135E-12 | 1.3999E-09 | 14/42 33.3% | 351/14524 2.4% |
| 31325 | positive regulation of cellular metabolic process | 5.73E-12 | 1.3999E-09 | 15/42 35.7% | 438/14524 3.0% |
| 9893 | positive regulation of metabolic process | 7.687E-12 | 1.4087E-09 | 15/42 35.7% | 447/14524 3.0% |
| 9891 | positive regulation of biosynthetic process | 1.091E-11 | 1.599E-09 | 14/42 33.3% | 371/14524 2.5% |
| 45941 | positive regulation of transcription | 2.004E-11 | 2.4478E-09 | 13/42 30.9% | 308/14524 2.1% |
| 10628 | positive regulation of gene expression | 2.664E-11 | 2.7892E-09 | 13/42 30.9% | 315/14524 2.1% |
| 45935 | positive regulation of nucleobase. nucleoside. nucleotide and nucleic acid metabolic process | 4.615E-11 | 4.2288E-09 | 13/42 30.9% | 329/14524 2.2% |
| 45893 | positive regulation of transcription. DNA-dependent | 7.408E-10 | 5.9212E-08 | 11/42 26.1% | 247/14524 1.7% |
| 51254 | positive regulation of RNA metabolic process | 8.078E-10 | 5.9212E-08 | 11/42 26.1% | 249/14524 1.7% |
| 51242 | positive regulation of cellular process | 1.016E-09 | 6.7667E-08 | 18/42 42.8% | 1018/14524 7.0% |
| 48518 | positive regulation of biological process | 5.13E-09 | 3.1334E-07 | 18/42 42.8% | 1124/14524 7.7% |
| 45595 | regulation of cell differentiation | 1.77E-08 | 9.9824E-07 | 9/42 21.4% | 180/14524 1.2% |
| 6357 | regulation of transcription from RNA polymerase II promoter | 2.371E-08 | 1.2414E-06 | 12/42 28.5% | 438/14524 3.0% |
| 45944 | positive regulation of transcription from RNA polymerase II promoter | 1.281E-07 | 6.2605E-06 | 8/42 19.0% | 156/14524 1.0% |
| 2376 | immune system process | 1.824E-07 | 8.3545E-06 | 14/42 33.3% | 777/14524 5.3% |
| 7275 | multicellular organismal development | 5.412E-07 | 0.000023336 | 21/42 50.0% | 2104/14524 14.4% |
| 48513 | organ development | 1.394E-06 | 0.000054211 | 15/42 35.7% | 1071/14524 7.3% |
| 10468 | regulation of gene expression | 1.405E-06 | 0.000054211 | 23/42 54.7% | 2680/14524 18.4% |
| 32502 | developmental process | 1.942E-06 | 0.000071168 | 23/42 54.7% | 2726/14524 18.7% |
| 30154 | cell differentiation | 4.198E-06 | 0.00014653 | 14/42 33.3% | 1000/14524 6.8% |
| 60255 | regulation of macromolecule metabolic process | 4.922E-06 | 0.00015756 | 23/42 54.7% | 2864/14524 19.7% |
| 1666 | response to hypoxia | 4.944E-06 | 0.00015756 | 5/42 11.9% | 55/14524 0.3% |
| 10556 | regulation of macromolecule biosynthetic process | 6.377E-06 | 0.00019477 | 22/42 52.3% | 2659/14524 18.3% |
| 43193 | positive regulation of gene-specific transcription | 7.311E-06 | 0.00021436 | 4/42 9.5% | 25/14524 0.1% |
| 9889 | regulation of biosynthetic process | 7.689E-06 | 0.00021677 | 22/42 52.3% | 2687/14524 18.5% |
| 30097 | hemopoiesis | 1.046E-05 | 0.00027865 | 6/42 14.2% | 118/14524 0.8% |
| 48869 | cellular developmental process | 1.066E-05 | 0.00027865 | 14/42 33.3% | 1080/14524 7.4% |
| 19222 | regulation of metabolic process | 1.102E-05 | 0.00027865 | 23/42 54.7% | 2991/14524 20.5% |
| 50793 | regulation of developmental process | 1.315E-05 | 0.00032122 | 12/42 28.5% | 780/14524 5.3% |
| 48534 | hemopoietic or lymphoid organ development | 2.017E-05 | 0.00046894 | 6/42 14.2% | 132/14524 0.9% |
| 6355 | regulation of transcription. DNA-dependent | 2.047E-05 | 0.00046894 | 20/42 47.6% | 2360/14524 16.2% |
| 48731 | system development | 2.214E-05 | 0.0004887 | 16/42 38.0% | 1518/14524 10.4% |
| 51252 | regulation of RNA metabolic process | 2.267E-05 | 0.0004887 | 20/42 47.6% | 2375/14524 16.3% |
| 2520 | immune system development | 2.504E-05 | 0.00051321 | 6/42 14.2% | 137/14524 0.9% |
| 35270 | endocrine system development | 2.63E-05 | 0.00051321 | 4/42 9.5% | 34/14524 0.2% |
| 32583 | regulation of gene-specific transcription | 2.63E-05 | 0.00051321 | 4/42 9.5% | 34/14524 0.2% |
| 45086 | positive regulation of interleukin-2 biosynthetic process | 2.661E-05 | 0.00051321 | 3/42 7.1% | 10/14524 0.0% |
| 31323 | regulation of cellular metabolic process | 3.188E-05 | 0.00059926 | 22/42 52.3% | 2913/14524 20.0% |
| 45449 | regulation of transcription | 5.716E-05 | 0.0010318 | 20/42 47.6% | 2517/14524 17.3% |
| 32501 | multicellular organismal process | 5.771E-05 | 0.0010318 | 23/42 54.7% | 3276/14524 22.5% |
| 31047 | gene silencing by RNA | 6.303E-05 | 0.0011 | 3/42 7.1% | 13/14524 0.0% |
| 2694 | regulation of leukocyte activation | 7.18E-05 | 0.0011968 | 5/42 11.9% | 94/14524 0.6% |
| 45076 | regulation of interleukin-2 biosynthetic process | 8.006E-05 | 0.0011968 | 3/42 7.1% | 14/14524 0.0% |
| 35195 | gene silencing by miRNA | 8.164E-05 | 0.0011968 | 2/42 4.7% | 2/14524 0.0% |
| 10039 | response to iron ion | 8.164E-05 | 0.0011968 | 2/42 4.7% | 2/14524 0.0% |
| 31050 | dsRNA fragmentation | 8.164E-05 | 0.0011968 | 2/42 4.7% | 2/14524 0.0% |
| 45368 | positive regulation of interleukin-13 biosynthetic process | 8.164E-05 | 0.0011968 | 2/42 4.7% | 2/14524 0.0% |
| 45366 | regulation of interleukin-13 biosynthetic process | 8.164E-05 | 0.0011968 | 2/42 4.7% | 2/14524 0.0% |
| 30918 | gene silencing by miRNA. production of miRNAs | 8.164E-05 | 0.0011968 | 2/42 4.7% | 2/14524 0.0% |
| 50865 | regulation of cell activation | 8.377E-05 | 0.0012028 | 5/42 11.9% | 97/14524 0.6% |
| 19219 | regulation of nucleobase. nucleoside. nucleotide and nucleic acid metabolic process | 8.533E-05 | 0.0012028 | 20/42 47.6% | 2582/14524 17.7% |
| 51244 | regulation of cellular process | 0.0001034 | 0.0014298 | 33/42 78.5% | 6568/14524 45.2% |
| 48856 | anatomical structure development | 0.0001203 | 0.0016324 | 16/42 38.0% | 1728/14524 11.8% |
| 46649 | lymphocyte activation | 0.0001415 | 0.0018864 | 5/42 11.9% | 108/14524 0.7% |
| 42110 | T cell activation | 0.0001989 | 0.0026039 | 4/42 9.5% | 56/14524 0.3% |
| 50791 | regulation of biological process | 0.0002101 | 0.0026972 | 33/42 78.5% | 6750/14524 46.4% |
| 45892 | negative regulation of transcription. DNA-dependent | 0.0002134 | 0.0026972 | 6/42 14.2% | 199/14524 1.3% |
| 51253 | negative regulation of RNA metabolic process | 0.0002322 | 0.0028443 | 6/42 14.2% | 202/14524 1.3% |
| 45082 | positive regulation of interleukin-10 biosynthetic process | 0.0002445 | 0.0028443 | 2/42 4.7% | 3/14524 0.0% |
| 45074 | regulation of interleukin-10 biosynthetic process | 0.0002445 | 0.0028443 | 2/42 4.7% | 3/14524 0.0% |
| 45404 | positive regulation of interleukin-4 biosynthetic process | 0.0002445 | 0.0028443 | 2/42 4.7% | 3/14524 0.0% |
| 45402 | regulation of interleukin-4 biosynthetic process | 0.0002445 | 0.0028443 | 2/42 4.7% | 3/14524 0.0% |
| 2504 | antigen processing and presentation of peptide or polysaccharide antigen via MHC class II | 0.0002884 | 0.0033033 | 3/42 7.1% | 21/14524 0.1% |
| 45321 | leukocyte activation | 0.0003599 | 0.0040582 | 5/42 11.9% | 131/14524 0.9% |
| 48523 | negative regulation of cellular process | 0.0003802 | 0.0041848 | 12/42 28.5% | 1084/14524 7.4% |
| 45580 | regulation of T cell differentiation | 0.0003825 | 0.0041848 | 3/42 7.1% | 23/14524 0.1% |
| 122 | negative regulation of transcription from RNA polymerase II promoter | 0.0004011 | 0.0043241 | 5/42 11.9% | 134/14524 0.9% |
| 50863 | regulation of T cell activation | 0.0004557 | 0.0048341 | 4/42 9.5% | 69/14524 0.4% |
| 19882 | antigen processing and presentation | 0.0004823 | 0.0048341 | 4/42 9.5% | 70/14524 0.4% |
| 16441 | posttranscriptional gene silencing | 0.000488 | 0.0048341 | 2/42 4.7% | 4/14524 0.0% |
| 35194 | posttranscriptional gene silencing by RNA | 0.000488 | 0.0048341 | 2/42 4.7% | 4/14524 0.0% |
| 32909 | regulation of transforming growth factor-beta2 production | 0.000488 | 0.0048341 | 2/42 4.7% | 4/14524 0.0% |
| 7183 | SMAD protein complex assembly | 0.000488 | 0.0048341 | 2/42 4.7% | 4/14524 0.0% |
| 48519 | negative regulation of biological process | 0.0006901 | 0.006745 | 12/42 28.5% | 1152/14524 7.9% |
| 16458 | gene silencing | 0.0007005 | 0.006756 | 3/42 7.1% | 28/14524 0.1% |
| 45619 | regulation of lymphocyte differentiation | 0.0007797 | 0.0074227 | 3/42 7.1% | 29/14524 0.1% |
| 65007 | biological regulation | 0.0008063 | 0.007577 | 33/42 78.5% | 7117/14524 49.0% |
| 1775 | cell activation | 0.0009891 | 0.0091772 | 5/42 11.9% | 162/14524 1.1% |
| 10605 | negative regulation of macromolecule metabolic process | 0.0010718 | 0.0097754 | 7/42 16.6% | 386/14524 2.6% |
| 51249 | regulation of lymphocyte activation | 0.0010802 | 0.0097754 | 4/42 9.5% | 86/14524 0.5% |
| 43331 | response to dsRNA | 0.0012156 | 0.010866 | 2/42 4.7% | 6/14524 0.0% |
| 2682 | regulation of immune system process | 0.0014604 | 0.012897 | 5/42 11.9% | 176/14524 1.2% |
| 16481 | negative regulation of transcription | 0.0014807 | 0.012921 | 6/42 14.2% | 282/14524 1.9% |
| 10629 | negative regulation of gene expression | 0.0015988 | 0.013787 | 6/42 14.2% | 286/14524 1.9% |
| 1934 | positive regulation of protein amino acid phosphorylation | 0.0016316 | 0.013906 | 3/42 7.1% | 37/14524 0.2% |
| 51869 | response to stimulus | 0.0016912 | 0.014249 | 18/42 42.8% | 2627/14524 18.0% |
| 45637 | regulation of myeloid cell differentiation | 0.0019114 | 0.015921 | 3/42 7.1% | 39/14524 0.2% |
| 31324 | negative regulation of cellular metabolic process | 0.0019727 | 0.016247 | 7/42 16.6% | 426/14524 2.9% |
| 9888 | tissue development | 0.0020721 | 0.016876 | 6/42 14.2% | 300/14524 2.0% |
| 9892 | negative regulation of metabolic process | 0.0021192 | 0.01707 | 7/42 16.6% | 431/14524 2.9% |
| 45622 | regulation of T-helper cell differentiation | 0.0022608 | 0.017819 | 2/42 4.7% | 8/14524 0.0% |
| 43370 | regulation of CD4-positive. alpha beta T cell differentiation | 0.0022608 | 0.017819 | 2/42 4.7% | 8/14524 0.0% |
| 42108 | positive regulation of cytokine biosynthetic process | 0.0023865 | 0.01861 | 3/42 7.1% | 42/14524 0.2% |
| 45934 | negative regulation of nucleobase. nucleoside. nucleotide and nucleic acid metabolic process | 0.0025167 | 0.019418 | 6/42 14.2% | 311/14524 2.1% |
| 42327 | positive regulation of phosphorylation | 0.0025604 | 0.019549 | 3/42 7.1% | 43/14524 0.2% |
| 10558 | negative regulation of macromolecule biosynthetic process | 0.0028366 | 0.021436 | 6/42 14.2% | 318/14524 2.1% |
| 45058 | T cell selection | 0.0029014 | 0.021701 | 2/42 4.7% | 9/14524 0.0% |
| 1655 | urogenital system development | 0.0029322 | 0.02171 | 3/42 7.1% | 45/14524 0.3% |
| 45937 | positive regulation of phosphate metabolic process | 0.0031304 | 0.022719 | 3/42 7.1% | 46/14524 0.3% |
| 10562 | positive regulation of phosphorus metabolic process | 0.0031304 | 0.022719 | 3/42 7.1% | 46/14524 0.3% |
| 35295 | tube development | 0.0032015 | 0.023007 | 4/42 9.5% | 114/14524 0.7% |
| 48732 | gland development | 0.0033372 | 0.023521 | 3/42 7.1% | 47/14524 0.3% |
| 30099 | myeloid cell differentiation | 0.0033372 | 0.023521 | 3/42 7.1% | 47/14524 0.3% |
| 9890 | negative regulation of biosynthetic process | 0.0035153 | 0.02454 | 6/42 14.2% | 331/14524 2.2% |
| 22415 | viral reproductive process | 0.0035525 | 0.024566 | 3/42 7.1% | 48/14524 0.3% |
| 21510 | spinal cord development | 0.0036201 | 0.024799 | 2/42 4.7% | 10/14524 0.0% |
| 48514 | blood vessel morphogenesis | 0.0037703 | 0.025397 | 4/42 9.5% | 119/14524 0.8% |
| 31401 | positive regulation of protein modification process | 0.0037766 | 0.025397 | 3/42 7.1% | 49/14524 0.3% |
| 30900 | forebrain development | 0.0042516 | 0.028331 | 3/42 7.1% | 51/14524 0.3% |
| 51094 | positive regulation of developmental process | 0.0052522 | 0.034683 | 6/42 14.2% | 357/14524 2.4% |
| 16032 | viral reproduction | 0.0059022 | 0.038628 | 3/42 7.1% | 57/14524 0.3% |
| 46637 | regulation of alpha-beta T cell differentiation | 0.0062404 | 0.039822 | 2/42 4.7% | 13/14524 0.0% |
| 21782 | glial cell development | 0.0062404 | 0.039822 | 2/42 4.7% | 13/14524 0.0% |
| 1568 | blood vessel development | 0.0062 | 0.0398 | 4/42 9.5% | 136/14524 0.9% |
| 7420 | brain development | 0.0066 | 0.0417 | 4/42 9.5% | 138/14524 0.9% |
| 1944 | vasculature development | 0.0068 | 0.0425 | 4/42 9.5% | 139/14524 0.9% |
| 45646 | regulation of erythrocyte differentiation | 0.0073 | 0.0451 | 2/42 4.7% | 14/14524 0.0% |
| 7167 | enzyme linked receptor protein signaling pathway | 0.0075 | 0.0470 | 5/42 11.9% | 253/14524 1.7% |

GO biological functions of validated targets of miRNAs overexpressed by MVs derived from MSCs, detected as % of cluster and total frequency of genes with a GO annotation. Only clusters with *p* value <0.05 are reported.
